# Diabetes and obesity in Down Syndrome across the lifespan: a retrospective cohort study using UK electronic health records

**DOI:** 10.2337/dc22-0482

**Published:** 2022-09-30

**Authors:** Aisha A Aslam, R Asaad Baksh, Sarah E Pape, Andre Strydom, Martin C Gulliford, Li F Chan, Go-DS21 consortium

**Affiliations:** 1 Centre for Endocrinology, William Harvey Research Institute, Barts and the London School of Medicine and Dentistry, Queen Mary University of London, London, UK; 2 Institute of Psychiatry, Psychology & Neuroscience, King’s College London, London, UK; 3 The LonDowns Consortium, London, UK; 4 South London and Maudsley NHS Foundation Trust, London, UK; 5 Department of Basic and Clinical Neuroscience, King’s College London, Institute of Psychiatry Psychology and Neuroscience, London, UK

**Keywords:** diabetes, obesity, Down syndrome

## Abstract

**Objective:**

Down Syndrome (DS) is the commonest form of chromosomal trisomy. Genetic factors in DS may increase the risk for diabetes. This study aimed to determine whether DS is associated with increased incidence of diabetes and the relationship with obesity across the lifespan compared to controls.

**Methods:**

Matched population-based cohort study (UK Clinical Practice Research Datalink, 1990-2020).

**Results:**

9,917 DS and 38,266 control patients were analysed. Diabetes rates were higher in DS individuals (incidence rate ratio 3.67; 95% CI 2.43 – 5.55; p<0.0001) and peaked at younger age; median age at diagnosis of 38 (28 to 49) years compared to 53 (43 to 61) years in controls. Incidence rates for T1DM were 0.44 per 1,000 patient years (0.31 to 0.61) in DS compared to 0.13 (0.09 to 0.17) in controls. T2DM rates were higher in DS compared to controls at age groups 5 years up to 34 years. In DS, peak mean BMI (kg/m^2^) was higher and at younger age (male=31.2 at 31 years; female=32.1 at 43 years) compared to controls (males=29.5 at 54 years; females 29.2 at 51 years); obesity was associated with an increased incidence of T2DM.

**Conclusions:**

At younger ages, the incidence of diabetes in DS patients is up to four times that of controls. Peak mean BMI is higher and established earlier in DS, contributing to T2DM risk. Further investigation into the relationship between obesity and diabetes in DS is required to inform treatment and prevention measures.

Down syndrome (DS) is the most common form of trisomy, birth anomaly and genetic cause of intellectual disability (ID).^1^ People with DS have a constellation of well recognised facial features as well as other medical conditions including congenital heart defects, gastrointestinal abnormalities, haematological abnormalities, and immune dysregulation caused by an extra copy of chromosome 21.^2^ The incidence of DS is estimated at 1 in 1000 births worldwide and in the UK.^3^

People with DS are at increased risk of type 1 diabetes mellitus (T1DM) compared to the general population. The increased incidence of T1DM is thought to be due to trisomy of genes on chromosome 21 and increased defects of the immune system in DS.^4,5^The gene for a transcriptional regulator, autoimmune regulatory protein (AIRE), is located on chromosome 21 and it is established that AIRE expression is reduced in DS patients. It is thought that this may promote autoimmunity in DS and thus the development of T1DM.^6^ Exact mechanisms are not clearly established, but it is suggested that mitochondrial dysfunction and increased oxidative stress are features of DS cells that may increase susceptibility to diabetes.^7^

Obesity and type 2 diabetes mellitus (T2DM) rates have increased in the general population but it is not known whether this similarly affected people with DS.^8–10^ Nonetheless, studies have shown that there is a higher incidence of obesity in DS which predisposes to insulin resistance.^11^ Furthermore, DS patients show an increased rate of non-alcoholic fatty liver disease (NAFLD), which is closely associated with insulin resistance.^12^

As well as obesity, a number of genes located on the DS critical region of chromosome 21, including Dual Specificity Tyrosine Phosphorylation Regulated Kinase 1A (DYRK1A) and Regulator Of Calcineurin 1 (RCAN1), are being investigated and thought to contribute to diabetes in DS.^13,14^ Loss of ß cell mass is a key feature in both T1DM and T2DM.^13,15^ Studies have shown that inhibition of DYRK1A induces ß cell proliferation.^16,17^ Wang *et al* further explored the role of DYRK1A as a regulator of proliferation in human ß cells and found that overexpression of DYRK1A attenuated ß cell proliferation in human islet cells and conversely reducing endogenous DYRK1A led to an increase in human ß cell proliferation.^17^ Similarly, rodent studies have found that overexpression of RCAN1 causes diabetes, age-associated hyperglycaemia, impaired glucose tolerance, hypoinsulinaemia, loss of ß cells and the down-regulation of key ß cell genes.^14^

To determine the natural history in a representative sample of people with DS across age ranges and trend over the decades of diabetes and obesity in DS, we performed a large retrospective cohort study using linked electronic health records. We examined diabetes risk, age of onset and obesity in DS in comparison to the general population over the last three decades using a nationally representative primary care database. Specifically, using this dataset we analysed the association of DS with diabetes and the relationship with obesity across the lifespan compared to population controls.

## Research Design and Methods

### Data source

We carried out a matched population-based cohort study in the UK Clinical Practice Research Datalink (CPRD) GOLD database employing data collected over 3 decades (between 1990 to 2020). The CPRD GOLD is one of the world’s largest databases of primary care electronic health records, with participation of about 7% of UK family practices and with ongoing collection of anonymised data from approximately 9 million patients.^18^ The high quality of CPRD GOLD data has been confirmed in many studies.^19^ The protocol was approved by the CPRD Independent Scientific Advisory Committee (ISAC protocol 20-048R).

All patients with DS were identified from the July 2020 release of CPRD GOLD using 10 Read codes for DS or Trisomy 21. Control participants were sampled from the list of all registered patients in the database without DS. Controls were exactly matched on family practice, gender and year of birth; and for calendar date of start of record to within 1.5 years. Up to four controls were randomly sampled for each DS participant using the ‘sample’ function in the R program.^20^

### Main measures

Participants were classified as having diabetes if a diabetes diagnosis was recorded in CPRD GOLD clinical or referral files (diagnostic code list in supplementary table 1), or if they were prescribed oral hypoglycaemic drugs or insulin (medication code list in supplementary table 2), or if they had a HbA1c≥48mmol/mol recorded on two or more occasions. The date of the first event of any type that was more than 365 days after the start of the patients’ registration was considered as the diabetes incidence date. In supplementary analyses, participants were classified as having T1DM if they were first prescribed insulin within 91 days of the diabetes diagnosis and were aged less than 35 years.^21^ Otherwise they were classified as T2DM. Records for body mass index (BMI) were analysed after calculating BMI from height and weight where appropriate. BMI records for adults aged 18 years and older were classified according to the WHO categories of ‘underweight’ (BMI <18.5 kg/m^2^), ‘normal’ (BMI 18.5-24.9 kg/m^2^), ‘overweight’ (BMI 25 – 29.9 kg/m^2^), or ‘obese’ (BMI ≥30 kg/m^2^). Children and young people aged between two and 17 years were grouped into the same categories using the international BMI standards^1,22^ using ‘zanthro’ package^23^ in Stata.^24^ The mean of values recorded in each year of age were employed for each patient. Overall mean BMI values were plotted by single year of age, only including mean values where there were five or more patients with observations, with no imputation. Since BMI was not recorded in every year, BMI categories were imputed using the method of last observation carried forwards, or backwards, allowing patients to remain in the same category for up to five years following a measurement.

### Analysis

In order to analyse changes in diabetes rates over time, incidence per 1,000 person years was used. Person time was analysed between 1990 and 2020. The start of each patient’s record was the latest of the patient registration date, the practice ‘up to standard date’ or the 1^st^ January 1990; while the end of record was the earliest of the end of registration, patient death date, last data collection date at the practice, or 1^st^ July 2020. Records of patients who developed diabetes were censored at the index date. New diagnoses of diabetes were compared for DS patients and controls by aggregating over age-group, sex and period. Age was divided into the categories 0 to 4 years, 5 to 14 years and then 10-year age groups up to 65 to 75 years. Periods were divided into 1990-9, 2000-9 and 2010-2020. Incidence rates were estimated per 1,000 person years, and confidence intervals were derived from the Poisson distribution. A Poisson regression model was fitted to calculate an adjusted incidence rate ratio (IRR). Age was fitted as a continuous predictor in the regression model, with a quadratic term to allow for non-linearity; similarly calendar year and year-squared were fitted. Sex and DS status were fitted as factors. An interaction term between DS and age was included. Predicted rates were plotted.

## Results

There were 10,247 patients who were diagnosed with DS and were registered in CPRD GOLD at any time between 1990 and 2020; 43 patients recorded with DS who reached ages over 75 were excluded because they were outliers in terms of typical DS life expectancy.^2^

This left 10,204 (5,372 female) patients with DS and 39,814 (20,681 female) control patients as comparators. There were 287 cases of pre-existing diabetes in DS patients and 433 in controls at cohort entry. As the study focuses on incidence (new onset) rates from birth-75 years of age, these pre-existing diabetes patients were excluded. After excluding these prevalent cases there were 9,917 DS patients eligible for the analysis of diabetes incidence. In the control group prevalent cases of diabetes were removed, as well as 1,115 controls who did not have comparator DS patients. Following these exclusions therefore, the final sample of controls eligible for analysis was 38,266.

There were 287 new diagnoses of diabetes in patients with DS and 1,254 in controls (Supplementary table 4). There were 83,160.5 person years of follow-up for patients with DS and 389,136.1 person years for controls (Table 1). Out of the 287 new cases in DS, 37 (12.9%) were T1DM and 250 (87.9%) were T2DM. In the control population, out of 1254 new cases, 49 (3.9%) were T1DM and 1205 (96.1%) were T2DM. There were similar proportions of male and female patients in both DS and control diabetes groups (Supplementary table 4).

### Changes through the decades

Cases of diabetes increased more than three times over the last three decades in both DS participants and the control population (Table 1). In the period 1990 – 1999, the incidence of diabetes per 1,000 patient years was 1.29 and 1.16 in DS patients and controls respectively. This increased to 3.26 in DS patients and 3.04 in population controls in the period 2000 – 2009 and increased further to 4.40 and 4.30 respectively in the period 2010 – 2020.

### Changes with age

**Figure 1:**
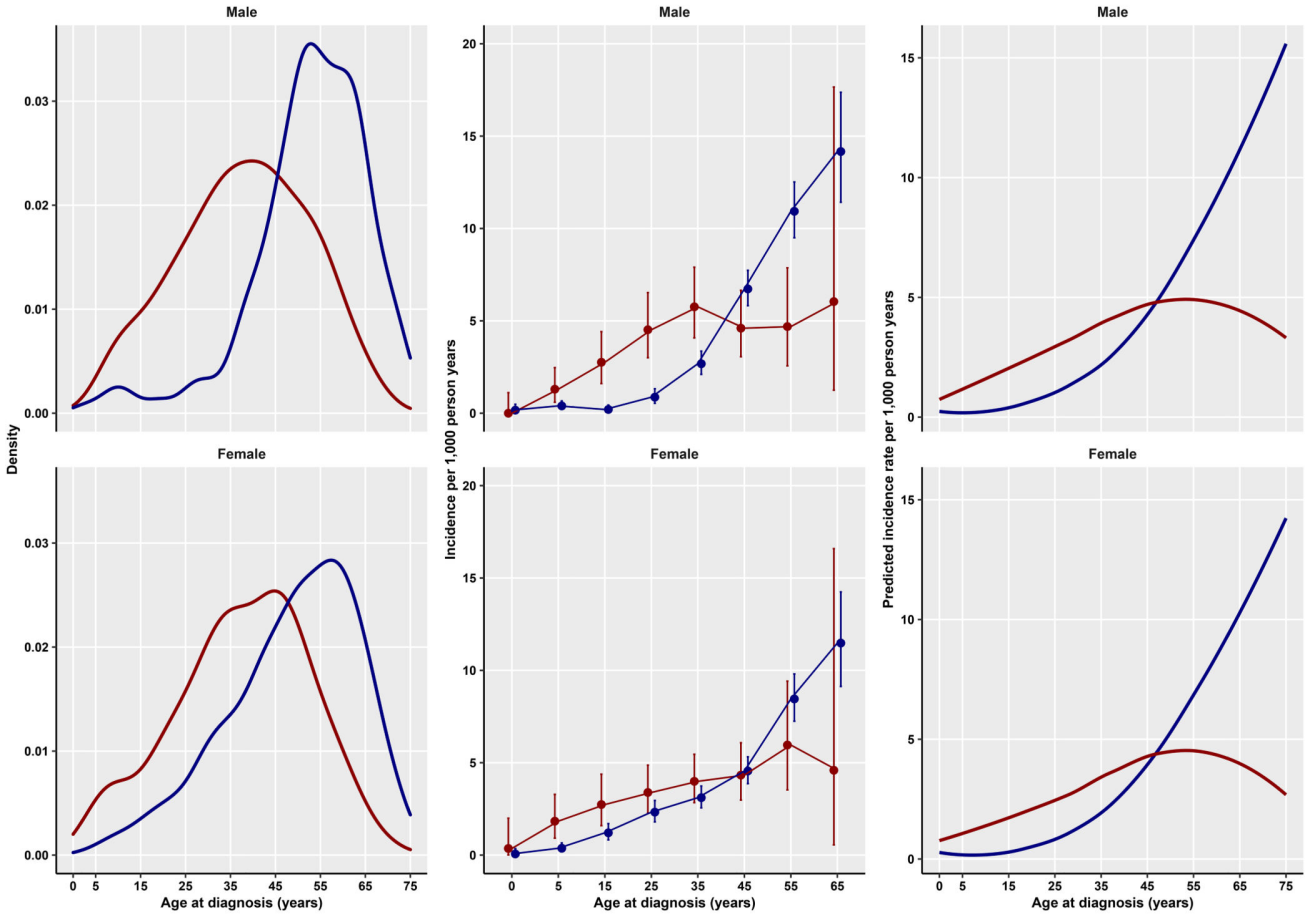
Distribution of age at diagnosis of diabetes by sex for patients with Down syndrome (red) and controls (blue). Left panel, density plot of incident cases; middle panel, age-specific incidence rates (95% confidence intervals); right panel, predicted incidence by age from regression model.

The median age (interquartile range) at diagnosis of diabetes was 38 (28 to 49) years in patients with DS compared to 53 (43 to 61) years in population controls (Supplementary table 4). Figure 1 shows the distribution of age at diagnosis of diabetes by sex for patients with DS (red) and controls (blue) with increased cases of diabetes in DS in both men and women compared to controls up to approximately 45 years old. In children and young adults, diabetes was four times more common in DS than in population controls (Table 1); incidence rate per 1,000 patient years (95% confidence interval) of 1.55 (0.95 to 2.39) in DS compared to 0.38 (0.25 to 0.57) in controls at ages 5 to 14 years and 2.75 (1.90 to 3.84) in DS compared to 0.68 (0.48 to 0.93) in controls at ages 15 to 24 years. The incidence of diabetes was approximately double in patients with DS aged 25 to 44 years; incidence rate per 1,000 patient years (95% confidence interval) of 3.86 (2.93 to 5.01) at 25 to 34 years compared to 1.65 (1.32 to 2.04) at 25 to 34 years and 4.70 (3.71 to 5.88) compared to 2.92 (2.52 to 3.37) at 35 to 44 years in DS patients compared to controls respectively. At 45 to 54 years old, the incidence rate was comparable in population controls compared to DS, and at older age groups, diabetes was approximately twice as common in population controls compared with DS patients.

### Incidence rate ratio for diabetes

After accounting for age, age/DS interaction, sex, calendar year and BMI (Table 2), the incidence of diabetes was nearly 4 times higher in people with DS compared to controls (IRR 3.67; 95% CI 2.43 – 5.55; p<0.0001). However, the effect declined with age (IRR 0.96, 95% CI 0.96 – 0.97; p<0.0001), and at older ages, people with DS had lower incidence rates of diabetes (figure 1).

### Type 1 and type 2 diabetes

The overall incidence rate per 1,000 patient years (95% confidence interval) of T1DM was over 3 times higher in patients with DS compared to population controls; 0.44 (0.31 to 0.61) compared to 0.13 (0.09 to 0.17) respectively (Supplementary table 5). 15 to 24 year old patients with DS had the highest incidence rate per 1,000 patient years of T1DM; 1.13 (0.62 to 1.90) in DS patients compared to 0.18 (0.09 to 0.33) in general population controls. At all other age groups (up to 34 years of age) there was no clear difference in the incidence of T1DM between groups (Supplementary table 5; supplementary figure 1).

Overall, the incidence of T2DM was similar between DS patients (3.01 per 1,000 patient years; 2.65 to 3.40) and population controls (3.10; 2.92 to 3.28) (Supplementary table 6). However, when broken down by age group, there was a much higher incidence of T2DM in DS patients compared to controls at age groups 5 years up to 34 years (Supplementary table 6, supplementary figure 2). At 5 to 14 years old, the incidence of T2DM was 10 times higher in patients with DS compared to controls; 0.62 per 1,000 patient years (0.27 to 1.22) compared to 0.06 (0.02 to 0.16) respectively (Supplementary table 6, supplementary figure 2). In both groups, incidence increased by age with a higher incidence in DS patients at age groups up until 45 years old. Rates were comparable between groups at age 45-54 years old. There was approximately double the incidence of T2DM in population controls compared to DS patients at age groups above 54 years old.

### Impact of obesity

**Figure 2:**
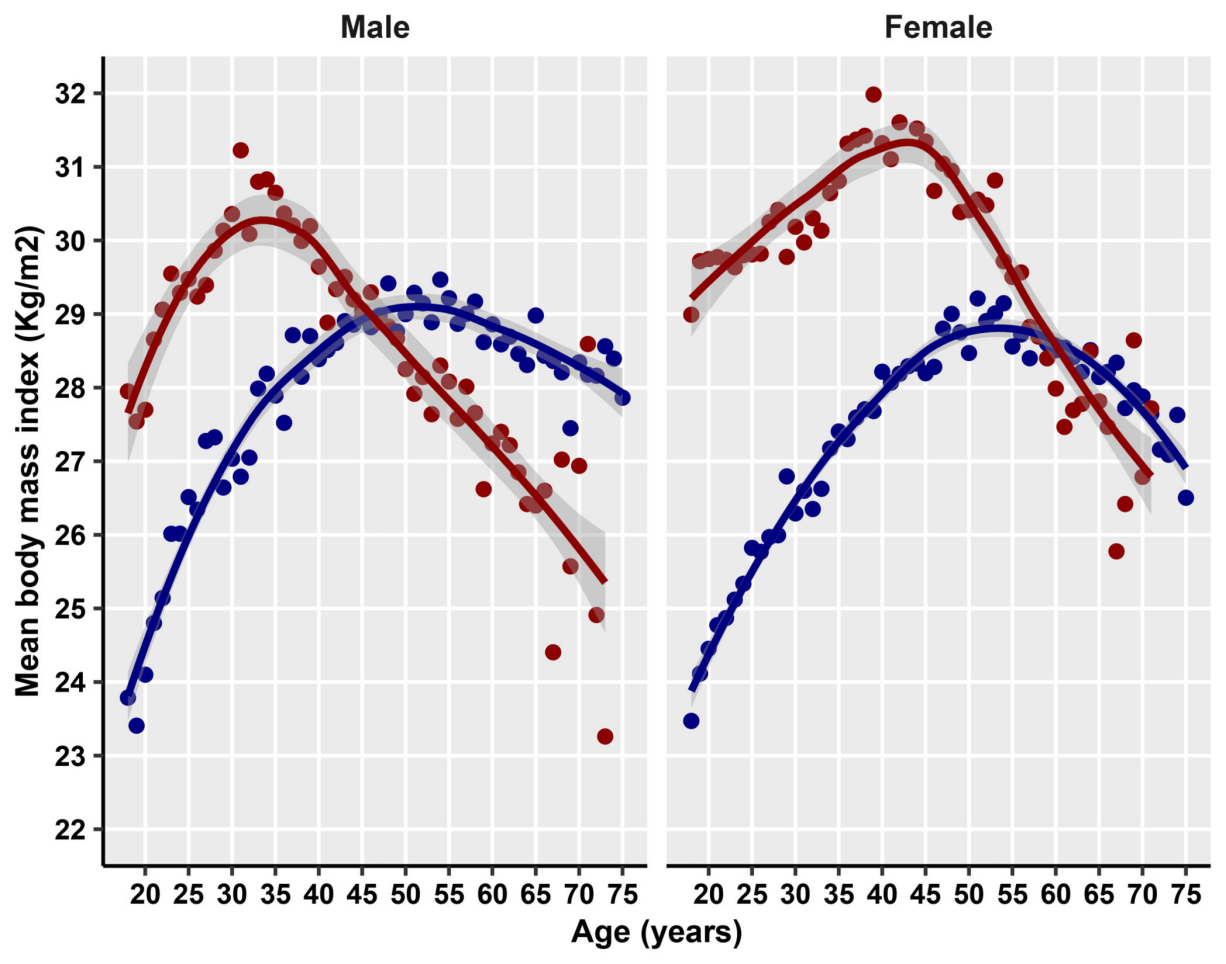
Mean body mass index (Kg/m^2^) by single year of age and sex for Down syndrome patients (red) and population controls (blue).

There was an overall increased risk of diabetes in overweight and obese patients compared to those with normal weight; incidence rate ratio (95% confidence interval) 1.92 (1.59 to 2.31) and 4.43 (3.74 to 5.25) respectively (Table 2). Mean (SD) BMI (kg/m^2^) was 34.3 (8.7) in DS patients and 33.2 (7.1) in controls (Supplementary table 4). Figure 2 shows increased mean BMI at up to approximately 45 years of age in men and 60 years of age in women with DS (red) compared to population controls (blue). Younger patients with DS have higher peaks in mean BMI compared to population controls as do women with DS compared to male counterparts and compared to general population controls (Figure 2). In DS, greatest mean BMI was reached at 31 years in males (31.2 Kg/m^2^) and 43 years in females (32.1 Kg/m^2^), compared with 54 years in male controls (29.5 Kg/m^2^) and 51 years in females (29.2 Kg/m^2^). In both groups, raised BMI was associated with increased incidence of diabetes. In DS patients, incidence of diabetes per 1,000 patient years was 8.07 (6.82 to 9.48) in patients with obesity compared to 4.23 (3.15 to 5.56) in patients who were overweight and 2.50 (1.70 to 3.55) in patients with normal weight (Table 1). In general population controls, the incidence of diabetes per 1,000 patient years was markedly higher with increasing BMI; 2.51 (2.10 to 2.97) in patients with normal weight, 7.29 (6.49 to 8.15) in patients who were overweight and 17.12 (15.86 to 18.46) in patients who were obese (Table 1).

There was no difference in incidence of T1DM by BMI category for either group (Supplementary table 5). There was an increased incidence of T2DM with increased BMI in both groups (Supplementary table 6). The incidence of T2DM per 1,000 patient years in DS rose from 1.93 (1.24 to 2.88) in patients with normal BMI to 3.90 (2.87 to 5.19) and 7.58 (6.37 to 8.95) in overweight and obese BMI categories respectively. In general population controls, the incidence per 1,000 patient years of T2DM rose with increased weight gain from 2.24 (1.86 to 2.68) in patients with normal BMI to 7.12 (6.34 to 7.98) and 16.89 (15.63 to 18.22) in overweight and obese BMI categories, respectively.

### Management

DS patients were less likely to be recorded as receiving oral hyperglycaemic medication within the first 5 years from diagnosis than controls (Supplementary table 4: 113 out of 287 (39%) patients with DS and 722 out of 1,254 (58%)). 58 (20%) patients with DS and 138 (11%) population controls were receiving insulin therapy. Median levels of HbA1c (53 mmol/mol) were similar between DS patients and controls.

## Discussion

In this UK based cohort study using a nationally representative primary care database, we show evidence of an increased incidence of diabetes over the last three decades from 1990 to 2020 in both patients with DS and population controls. This is in keeping with global and UK based studies which have shown increasing incidence of T2DM across the population up until approximately the mid 2000s with stable or falling rates in subsequent years.^8^

National surveys in the UK show clear increase in the proportion of overweight or obese adults between 1993 and 2001 with small changes since then.^9^ Similarly, surveys of children show an increase in obesity between 1995 to 2004 with subsequent broadly steady incidence of obesity in children.^10^ Obesity is a recognised risk factor in other diseases with clear associations between obesity and T2DM and coronary heart disease.^25,26^ Studies have shown increased incidence of overweight and obesity in both adults and children with DS.^27,28^

Our study shows an adjusted increased incidence rate ratio of 3.67 (95% confidence interval 2.43 to 5.55) of diabetes in DS. There is a difference in the age of onset of diabetes between the populations, with incidence in DS increased in age groups below 44 years. When we examined age group specific incidence, our study showed that people with DS are at significantly increased risk of diabetes at a younger age compared to the general population with over four times the risk in children and young adults (aged 5 to 24 years) and over double the risk in patients aged 25 to 44 years old. We show that there is an increased risk for T1DM in children and young people with DS compared to population controls with almost three times the risk in children aged 5 to 14 years old and seven times the risk in patients aged 15 to 24 years old. The incidence for T2DM is over 10 times higher in children aged 5 to 14 years with DS compared to controls. There remains an increased incidence for T2DM (although less profound a difference) in age groups up to 45 years old. In older age groups above 55 years old, there is an increased incidence of T2DM in general population controls compared to DS patients.

Our study also reiterates that raised BMI is associated with increased incidence of diabetes and additionally shows that in patients with DS, obesity affects younger age groups. We show that being overweight confers an increased risk of T2DM in both groups and that this additional risk is higher in general population controls compared to DS patients.

Underlying mechanisms for this increased susceptibility for diabetes in DS still need further investigation. A combination of factors including genetic susceptibility, predisposition to autoimmunity, mitochondrial dysfunction, increased oxidative stress and cellular dysfunction are thought to contribute to this risk. Candidate genes include AIRE, DYRK1A and RCAN1.^6,14,16,17^ Some of the increased risk of diabetes at a younger age in DS may be explained by a predisposition to autoimmune conditions, more specifically of T1DM, in these patients. DS may be associated with a reduction in β cell mass and impaired insulin secretion in DS.^13–17^

In support of these different mechanisms, we found evidence for an increase in both T1DM and T2DM in patients with DS. Furthermore, we have shown that individuals with DS have a similar increase in diabetes incidence over time, thus are also subject to the environmental factors related to this increase, such as increased rates of obesity, and dietary risk factors. However, the extent to which obesity predisposes to diabetes in people with DS is somewhat reduced compared to the general population, suggesting that additional biological mechanisms associated with DS may be related to increased incidence of diabetes. Our study supports the theory for an underlying genetic predisposition to diabetes in patients with DS that may be additional to their risk of obesity.

HbA1c is a marker for glycaemic control and has been shown in some studies to correlate with risk of diabetic complications.^29^ Reassuringly, our analysis found similar median levels of HbA1c between DS patients and population controls.

### Limitations and methodology of the study

In the UK, primary care is the usual first point of contact for adult patients with T2DM and repeat prescriptions for both adults and children with diabetes of any kind are issued by primary care. In this way, UK CPRD is likely to better represent burden of disease compared to secondary care records. However, T1DM in adults and diabetes of any type in children is managed in secondary care, though with input from primary care; nevertheless, the CPRD database might underestimate the true incidence of T1DM. Furthermore, in contrast to adults with DS, annual health reviews in children with DS are usually performed by community paediatricians (in secondary care) with differing access to primary care health records. This is likely to be reflected in our study with missing BMI data for children with DS.

Our classification of T1DM (patients who were first prescribed insulin within 91 days of the diabetes diagnosis and were aged less than 35 years) may include patients with very poorly controlled diabetes of other types who may require insulin therapy earlier on, therefore misclassifying these cases as T1DM. Other published works using similar large clinical datasets also use non-standard definitions of T1DM and T2DM to reduce missing cases due to incomplete coding.^30–33^ Since we have been consistent in our definitions between our control group and DS group, we feel that the analyses nevertheless show a valuable difference between population groups.

In this study we classify diabetes in DS as T1DM and T2DM. We recognise there are some classification systems such as The international society for pediatric and adolescent diabetes (ISPAD) that would classify diabetes in DS under the alternative heading “other genetic syndromes sometimes associated with diabetes”.^34^ However this is not the case for classification systems such as The international statistical classification of diseases and related health problems (10^th^ revision) (ICD-10) where diabetes in DS would fall under T1DM and T2DM categories.^35^ Furthermore, in practice, clinicians caring for DS patients with diabetes would generally classify diabetes as T1DM and T2DM based on clinical and biochemical phenotype, which has direct implications in understanding of mechanisms as well as clinical management and prevention. Importantly, within the DS field it is accepted to classify diabetes as T1DM and T2DM as evidenced by previous publications including the DS Medical Interest Group.^4,36–38^ We acknowledge that the differentiation between T1DM and T2DM may require additional biochemical investigations which may not always be possible in the primary care setting. Finally, our paper reports on overall incidence of diabetes in DS compared to controls which includes all classifications both T1DM and T2DM as well as “other” types of diabetes (see supplementary table 1). In this way, our data is likely to better capture incidence of diabetes and reduce classification errors.

## Conclusion

The median age of diabetes diagnosis was 15 years earlier in DS and is over four times more common in children and young adults with DS compared to population controls. There is also an increased incidence of obesity in children and young adults with DS, with increasing rates over time. Our study suggests the need for close monitoring and early identification of diabetes (and obesity) in this susceptible population. Health promotion is vital in the prompt detection and treatment of T1DM and annual health checks for children with DS should ensure that those with parental responsibility are given adequate information of signs and symptoms of T1DM (namely polyuria, polydipsia and weight loss). HbA1c is not routinely measured in children and adolescents with DS. Our study suggests that they have an additional risk of T2DM and are also susceptible to the environmental factors driving increased rates of obesity and T2DM, and we would therefore recommend proactive monitoring of HbA1c at the annual health check in adolescents with DS. Furthermore, exploration of the genetic predisposition to diabetes and obesity in DS is necessary for development of effective therapies and to inform future preventive measures.

## Supplementary Material

Supplemental Material

## Figures and Tables

**Table 1:** Incidence of diabetes by sex, age-group, period and body mass index category for Down syndrome cases and population controls.

		Down Syndrome			Population Controls		
		Diabetes diagnoses	Person years at risk	Incidence per 1,000 patient years (95% confidence interval)	Diabetes diagnoses	Person years at risk	Incidence per 1,000 patient years (95% confidence interval)
**Total**		287	83,160.5	3.45 (3.06 to 3.87)	1254	389,136.1	3.22 (3.05 to 3.41)

**Period**	1990-9	15	11,602.1	1.29 (0.72 to 2.13)	71	60,947.0	1.16 (0.91 to 1.47)
	2000-9	122	37,480.8	3.26 (2.70 to 3.89)	550	180,986.6	3.04 (2.79 to 3.30)
	2010-20	150	34,077.6	4.40 (3.73 to 5.17)	633	147,202.5	4.30 (3.97 to 4.65)

**Sex**	Male	137	38,770.7	3.53 (2.97 to 4.18)	617	190,023.8	3.25 (3.00 to 3.51)
	Female	150	44,389.8	3.38 (2.86 to 3.97)	637	199,112.3	3.20 (2.96 to 3.46)

**Age-group**	0-4	1	6,104.5	0.16 (0.00 to 0.91)	4	33,986.7	0.12 (0.03 to 0.30)
	5-14	20	12,916.8	1.55 (0.95 to 2.39)	25	65,148.9	0.38 (0.25 to 0.57)
	15-24	34	12,379.2	2.75 (1.90 to 3.84)	38	56,126.1	0.68 (0.48 to 0.93)
	25-34	57	14,750.9	3.86 (2.93 to 5.01)	87	52,598.1	1.65 (1.32 to 2.04)
	35-44	77	16,366.0	4.70 (3.71 to 5.88)	186	63,677.5	2.92 (2.52 to 3.37)
	45-54	61	13,704.9	4.45 (3.40 to 5.72)	356	64,138.6	5.55 (4.99 to 6.16)
	55-64	32	6,006.0	5.33 (3.64 to 7.52)	384	39,821.9	9.64 (8.70 to 10.66)
	65-75	5	932.1	5.36 (1.74 to 12.52)	174	13,638.2	12.76 (10.93 to 14.80)

**BMI category**	Underweight	4	895.5	4.47 (1.22 to 11.44)	2	4,450.8	0.45 (0.05 to 1.62)
	Normal weight	31	12,408.2	2.50 (1.70 to 3.55)	133	53,086.0	2.51 (2.10 to 2.97)
	Overweight	51	12,053.3	4.23 (3.15 to 5.56)	306	41,988.1	7.29 (6.49 to 8.15)
	Obese	148	18,334.7	8.07 (6.82 to 9.48)	678	38,601.2	17.12 (15.86 to 18.46)
	Not recorded	53	39,468.8	1.34 (1.01 to 1.76)	135	250,010.0	0.54 (0.45 to 0.64)

**Table 2:** Results of a Poisson regression model. Diabetes incidence rate ratios were adjusted for each of the variables shown.

Variable	Category	Incidence rate ratio	95% confidence interval		P value
			LL	UL	
**Down syndrome**		3.67	2.43	5.55	0.000
**Calendar year (per year)**		1.09	1.04	1.13	0.000
**Calendar year-squared**		1.00	1.00	1.00	0.001
**Age (per year)**		1.06	1.04	1.08	0.000
**Age-squared**		1.00	1.00	1.00	0.031
**Down syndrome x age interaction**		0.96	0.96	0.97	0.000

**BMI category**	Not recorded	0.32	0.26	0.40	0.000
	Underweight	0.60	0.26	1.35	0.22
	Normal weight	Ref.			
	Overweight	1.92	1.59	2.31	0.000
	Obese	4.43	3.74	5.25	0.000

**Sex**	Male	Ref.			
	Female	0.76	0.69	0.84	0.000
