# Supplementary material for: Diabetes and obesity in Down Syndrome across the lifespan: a retrospective cohort study using UK electronic health records
Source: Diabetes Care. Author manuscript; Available in PMC 2022 Dec 1. (PMC7613880; doi:10.2337/dc22-0482)
Supplement: Supplemental Material [file EMS156840-supplement-Supplemental_Material.docx]

**Supplementary Table 1: Diagnostic Codes**

| **MEDCODE** | **READCODE** | **READTERM** |
| --- | --- | --- |
| 6813 | 1434 | H/O: diabetes mellitus |
| 30127 | 1435 | H/O: Admission in last year for hyperglycaemic disorder |
| 17236 | 14P3.00 | H/O: insulin therapy |
| 11050 | 44Uz.11 | Blood hyperglycaemia NOS |
| 27573 | 44Uz.00 | Blood glucose raised NOS |
| 19781 | 44V2.00 | Glucose tol. test impaired |
| 32770 | 44V3.00 | Glucose tol. test diabetic |
| 11599 | 7276 | Pan retinal photocoagulation for diabetes |
| 47341 | 8A12.00 | Diabetic crisis monitoring |
| 24363 | 8A13.00 | Diabetic stabilisation |
| 105574 | 8A17000 | Frequency of blood glucose self-monitoring |
| 55140 | 8A1A.00 | Self monitoring urine ketones |
| 17478 | 8A17.00 | Self monitoring of blood glucose |
| 17846 | 8A18.00 | Self monitoring of urine glucose |
| 42217 | 8A19.00 | Self monitoring of blood and urine glucose |
| 11471 | 8B3l.00 | Diabetes medication review |
| 103762 | 8BAi.00 | Insulin passport completed |
| 104858 | 8BAm.00 | Insulin passport checked |
| 108655 | 8BAp.00 | Insulin passport not checked |
| 12213 | 8BL2.00 | Patient on maximal tolerated therapy for diabetes |
| 8414 | 8CA4100 | Pt advised re diabetic diet |
| 12483 | 8CAQ.00 | Advice about blood glucose control |
| 103772 | 8CE0200 | Insulin passport given |
| 103816 | 8CE0100 | Insulin alert patient information booklet given |
| 18066 | 8CE0.00 | Diabetic leaflet given |
| 105585 | 8CMW700 | Diabetes clinical pathway |
| 28856 | 8CP2.00 | Transition of diabetes care options discussed |
| 63412 | 8CR2.00 | Diabetes clinical management plan |
| 47032 | 8CS0.00 | Diabetes care plan agreed |
| 113525 | 8CdD.00 | Advised to maintain back up supply insulin subcut injection |
| 7059 | 8H2J.00 | Admit diabetic emergency |
| 35321 | 8H3O.00 | Non-urgent diabetic admission |
| 7777 | 8H4F.00 | Referral to diabetologist |
| 12225 | 8H7C.00 | Refer, diabetic liaison nurse |
| 8306 | 8H7f.00 | Referral to diabetes nurse |
| 11677 | 8H7r.00 | Refer to diabetic foot screener |
| 18662 | 8HBH.00 | Diabetic retinopathy 6 month review |
| 11018 | 8HBG.00 | Diabetic retinopathy 12 month review |
| 57723 | 8HHy.00 | Referral to diabetic register |
| 61557 | 8HKE.00 | Diabetology D.V. requested |
| 47370 | 8HLE.00 | Diabetology D.V. done |
| 72333 | 8HME.00 | Listed for Diabetology admissn |
| 105207 | 8HTE100 | Referral to community diabetes clinic |
| 19381 | 8HTk.00 | Referral to diabetic eye clinic |
| 34541 | 8HVU.00 | Private referral to diabetologist |
| 47058 | 8Hg4.00 | Discharged from care of diabetes specialist nurse |
| 100422 | 8HgC.00 | Discharged from diabetes shared care programme |
| 109806 | 8Hgd.00 | Discharge from secondary care diabetes service |
| 47011 | 8Hj0.00 | Referral to diabetes structured education programme |
| 93870 | 8Hj5.00 | Referral to XPERT diabetes structured education programme |
| 93657 | 8Hj4.00 | Referral to DESMOND diabetes structured education programme |
| 93704 | 8Hj3.00 | Referral to DAFNE diabetes structured education programme |
| 82474 | 8Hl4.00 | Referral to community diabetes specialist nurse |
| 102389 | 8HlS.00 | Referral for management of impaired glucose tolerance |
| 94330 | 8H4e.00 | Referral to diabetes special interest general practitioner |
| 104287 | 8Hlc.00 | Referral to community diabetes service |
| 64142 | 8Hl1.00 | Referral for diabetic retinopathy screening |
| 18824 | 8I3W.00 | Diabetic foot examination declined |
| 12262 | 8I3X.00 | Diabetic retinopathy screening refused |
| 58159 | 8I3k.00 | Insulin therapy declined |
| 58639 | 8I57.00 | Patient held diabetic record declined |
| 95093 | 8I83.00 | Did not complete DESMOND diabetes structured educat program |
| 94956 | 8I84.00 | Did not complete XPERT diabetes structured education program |
| 97809 | 8I82.00 | Did not complete DAFNE diabetes structured education program |
| 95094 | 8I81.00 | Did not complete diabetes structured education programme |
| 107414 | 8I94.00 | Diabetes structured education programme not available |
| 101456 | 8IAs.00 | Diabetic dietary review declined |
| 103743 | 8IE2.00 | Diabetes care plan declined |
| 105937 | 8IEQ.00 | Referral to community diabetes specialist nurse declined |
| 106953 | 8IEa.00 | Referral to DAFNE diabetes structured educn prog declined |
| 20900 | 9OLA.11 | Diabetes monitored |
| 93854 | 9OLM.00 | Diabetes structured education programme declined |
| 31240 | 9OL7.00 | Diabetes monitor.verbal invite |
| 111649 | 9OLP.00 |  |
| 12030 | 9OL6.00 | Diabetes monitoring 3rd letter |
| 35383 | 9OLD.00 | Diabetic patient unsuitable for digital retinal photography |
| 13195 | 9OL5.00 | Diabetes monitoring 2nd letter |
| 93491 | 9OLJ.00 | DAFNE diabetes structured education programme completed |
| 26605 | 9OLB.00 | Attended diabetes structured education programme |
| 13194 | 9OL4.00 | Diabetes monitoring 1st letter |
| 22130 | 9OL3.00 | Diabetes monitoring default |
| 13197 | 9OL1.00 | Attends diabetes monitoring |
| 31141 | 9OL8.00 | Diabetes monitor.phone invite |
| 94011 | 9OLG.00 | Attended XPERT diabetes structured education programme |
| 93631 | 9OLL.00 | XPERT diabetes structured education programme completed |
| 26603 | 9OL2.00 | Refuses diabetes monitoring |
| 13191 | 9OL..11 | Diabetes clinic administration |
| 93530 | 9OLE.00 | Attended DESMOND structured programme |
| 51066 | 9OLC.00 | Family/carer attended diabetes structured education prog |
| 13192 | 9OLA.00 | Diabetes monitor. check done |
| 93390 | 9OLH.00 | Attended DAFNE diabetes structured education programme |
| 94186 | 9OLF.00 | Diabetes structured education programme completed |
| 31241 | 9OLZ.00 | Diabetes monitoring admin.NOS |
| 54846 | 9OL9.00 | Diabetes monitoring deleted |
| 93529 | 9OLK.00 | DESMOND diabetes structured education programme completed |
| 101455 | 9OLN.00 | Diabetes monitor invitation by SMS (short message service) |
| 9897 | 9OL..00 | Diabetes monitoring admin. |
| 106738 | 9Oy0000 | Diabetic foot screening invitation |
| 106722 | 9Oy0300 | Diabetic foot screening invitation second letter |
| 106723 | 9Oy0200 | Diabetic foot screening invitation first letter |
| 107793 | 9Oy0400 | Diabetic foot screening invitation third letter |
| 95124 | 9Oy0.00 | Diabetes screening invitation |
| 94647 | 9Oy..00 | Diabetes screening administration |
| 96143 | 9kL..00 | Insulin initiation - enhanced services administration |
| 110056 | 9m0B.00 | Excluded frm diab retinop screen as no currnt contct details |
| 107597 | 9m0D.00 | Excluded from diabetic retinopthy screen as learn disability |
| 106350 | 9m05.00 | Excluded from diabetic retinopathy screening as moved away |
| 106332 | 9m00.00 | Eligible for diabetic retinopathy screening |
| 109520 | 9m03.00 | Eligibility permanently inactive for diabetic retinop screen |
| 109521 | 9m02.00 | Eligibility temporarily inactive for diabetic retinop screen |
| 106328 | 9m07.00 | Excluded diabetc retinop screen as under care ophthalmolgist |
| 106218 | 9m0A.00 | Declined diabetic retinopathy screening |
| 106329 | 9m08.00 | Excluded from diabetic retinopathy screening as blind |
| 106351 | 9m09.00 | Excluded from diabetic retinop screen as no longer diabetic |
| 106327 | 9m04.00 | Excluded from diabetic retinopathy screening |
| 106352 | 9m06.00 | Excluded from diabetic retinopathy screening as deceased |
| 106778 | 9m0C.00 | Excluded frm diabetic retinopathy screen as terminal illness |
| 106445 | 9m0E.00 | Excluded from diabetic retinopathy screen physical disorder |
| 106269 | 9m0..00 | Diabetic retinopathy screening administrative status |
| 106273 | 9m90000 | Impaired glucose tolerance monitoring invitation 1st letter |
| 106220 | 9m9..00 | Impaired glucose tolerance monitoring administration |
| 106323 | 9m90200 | Impaired glucose tolerance monitoring invitation 3rd letter |
| 106275 | 9m90100 | Impaired glucose tolerance monitoring invitation 2nd letter |
| 106316 | 9m90.00 | Impaired glucose tolerance monitoring invitation |
| 91943 | C10EC11 | Type I diabetes mellitus with polyneuropathy |
| 101311 | C10EC12 | Insulin dependent diabetes mellitus with polyneuropathy |
| 56885 | C10K000 | Type A insulin resistance without complication |
| 64668 | C10FJ11 | Insulin treated Type II diabetes mellitus |
| 106061 | C10FP11 | Type II diabetes mellitus with ketoacidotic coma |
| 13279 | C104y00 | Other specified diabetes mellitus with renal complications |
| 36633 | C109K00 | Hyperosmolar non-ketotic state in type 2 diabetes mellitus |
| 52236 | C10A.00 | Malnutrition-related diabetes mellitus |
| 17262 | C109600 | Non-insulin-dependent diabetes mellitus with retinopathy |
| 18642 | C10EH00 | Type 1 diabetes mellitus with arthropathy |
| 47649 | C10E100 | Type 1 diabetes mellitus with ophthalmic complications |
| 62674 | C10FA00 | Type 2 diabetes mellitus with mononeuropathy |
| 95992 | C108A11 | Type I diabetes mellitus without complication |
| 42831 | C10E200 | Type 1 diabetes mellitus with neurological complications |
| 711 | C10..00 | Diabetes mellitus |
| 61071 | C109D12 | Type 2 diabetes mellitus with hypoglycaemic coma |
| 10692 | C10EM00 | Type 1 diabetes mellitus with ketoacidosis |
| 69993 | C10E600 | Type 1 diabetes mellitus with gangrene |
| 26054 | C10FL00 | Type 2 diabetes mellitus with persistent proteinuria |
| 47582 | C10E000 | Type 1 diabetes mellitus with renal complications |
| 93878 | C10E511 | Type I diabetes mellitus with ulcer |
| 97849 | C10E912 | Insulin dependent diabetes maturity onset |
| 39317 | C106100 | Diabetes mellitus, adult onset, + neurological manifestation |
| 37806 | C10FF00 | Type 2 diabetes mellitus with peripheral angiopathy |
| 108005 | C109312 | Type 2 diabetes mellitus with multiple complications |
| 45491 | C10z.00 | Diabetes mellitus with unspecified complication |
| 10098 | C10yy00 | Other specified diabetes mellitus with other spec comps |
| 99231 | C108B11 | Type I diabetes mellitus with mononeuropathy |
| 98616 | C10F211 | Type II diabetes mellitus with neurological complications |
| 66145 | C10EN11 | Type I diabetes mellitus with ketoacidotic coma |
| 44260 | C108F00 | Insulin dependent diabetes mellitus with diabetic cataract |
| 44443 | C108500 | Insulin dependent diabetes mellitus with ulcer |
| 56448 | C108A00 | Insulin-dependent diabetes without complication |
| 40401 | C109500 | Non-insulin dependent diabetes mellitus with gangrene |
| 49074 | C10F400 | Type 2 diabetes mellitus with ulcer |
| 61523 | C106y00 | Other specified diabetes mellitus with neurological comps |
| 65062 | C103z00 | Diabetes mellitus NOS with ketoacidotic coma |
| 42505 | C101z00 | Diabetes mellitus NOS with ketoacidosis |
| 12455 | C10E.11 | Type I diabetes mellitus |
| 98392 | C10C.12 | Maturity onset diabetes in youth type 1 |
| 46624 | C10C.11 | Maturity onset diabetes in youth |
| 1407 | C10FJ00 | Insulin treated Type 2 diabetes mellitus |
| 63357 | C107100 | Diabetes mellitus, adult, + peripheral circulatory disorder |
| 61344 | C108011 | Type I diabetes mellitus with renal complications |
| 21983 | C108012 | Type 1 diabetes mellitus with renal complications |
| 108360 | C10P000 | Type I diabetes mellitus in remission |
| 35399 | C107.00 | Diabetes mellitus with peripheral circulatory disorder |
| 113115 | C10Q.00 | Maturity onset diabetes of the young type 5 |
| 18683 | C10E500 | Type 1 diabetes mellitus with ulcer |
| 18278 | C109J00 | Insulin treated Type 2 diabetes mellitus |
| 43857 | C10M.00 | Lipoatrophic diabetes mellitus |
| 50527 | C10FB11 | Type II diabetes mellitus with polyneuropathy |
| 114439 | C10A200 | Malnutrition-related diabetes mellitus with renal complicatn |
| 99716 | C10EE12 | Insulin dependent diabetes mellitus with hypoglycaemic coma |
| 107701 | C10FK11 | Hyperosmolar non-ketotic state in type II diabetes mellitus |
| 11551 | C10B.00 | Diabetes mellitus induced by steroids |
| 22487 | C10N.00 | Secondary diabetes mellitus |
| 60499 | C108600 | Insulin dependent diabetes mellitus with gangrene |
| 758 | C10F.00 | Type 2 diabetes mellitus |
| 36695 | C10D.00 | Diabetes mellitus autosomal dominant type 2 |
| 24693 | C109G00 | Non-insulin dependent diabetes mellitus with arthropathy |
| 33969 | C10A100 | Malnutrition-related diabetes mellitus with ketoacidosis |
| 59903 | C106.11 | Diabetic amyotrophy |
| 7795 | C106.12 | Diabetes mellitus with neuropathy |
| 16491 | C106.13 | Diabetes mellitus with polyneuropathy |
| 46301 | C10EC00 | Type 1 diabetes mellitus with polyneuropathy |
| 51756 | C10FP00 | Type 2 diabetes mellitus with ketoacidotic coma |
| 18390 | C10FM00 | Type 2 diabetes mellitus with persistent microalbuminuria |
| 10418 | C10ED00 | Type 1 diabetes mellitus with nephropathy |
| 62352 | C108H11 | Type I diabetes mellitus with arthropathy |
| 58604 | C109611 | Type II diabetes mellitus with retinopathy |
| 42762 | C109612 | Type 2 diabetes mellitus with retinopathy |
| 64357 | C10zz00 | Diabetes mellitus NOS with unspecified complication |
| 95351 | C10FA11 | Type II diabetes mellitus with mononeuropathy |
| 95539 | C10FS00 | Maternally inherited diabetes mellitus |
| 102112 | C10E611 | Type I diabetes mellitus with gangrene |
| 109051 | C10E612 | Insulin dependent diabetes mellitus with gangrene |
| 68792 | C10z000 | Diabetes mellitus, juvenile type, + unspecified complication |
| 56268 | C109D11 | Type II diabetes mellitus with hypoglycaemic coma |
| 64446 | C108G00 | Insulin dependent diab mell with peripheral angiopathy |
| 62613 | C10EA11 | Type I diabetes mellitus without complication |
| 24694 | C108B00 | Insulin dependent diabetes mellitus with mononeuropathy |
| 34268 | C10F200 | Type 2 diabetes mellitus with neurological complications |
| 34912 | C109400 | Non-insulin dependent diabetes mellitus with ulcer |
| 93922 | C104000 | Diabetes mellitus, juvenile type, with renal manifestation |
| 25591 | C10FQ00 | Type 2 diabetes mellitus with exudative maculopathy |
| 47954 | C10F900 | Type 2 diabetes mellitus without complication |
| 51957 | C108511 | Type I diabetes mellitus with ulcer |
| 38617 | C101y00 | Other specified diabetes mellitus with ketoacidosis |
| 65025 | C107z00 | Diabetes mellitus NOS with peripheral circulatory disorder |
| 111106 | C108A12 | Type 1 diabetes mellitus without complication |
| 34283 | C105z00 | Diabetes mellitus NOS with ophthalmic manifestation |
| 70448 | C107000 | Diabetes mellitus, juvenile +peripheral circulatory disorder |
| 107824 | C10P100 | Type II diabetes mellitus in remission |
| 46850 | C108811 | Type I diabetes mellitus - poor control |
| 45914 | C108812 | Type 1 diabetes mellitus - poor control |
| 98723 | C10FD11 | Type II diabetes mellitus with hypoglycaemic coma |
| 107603 | C10P.00 | Diabetes mellitus in remission |
| 102163 | C10ED12 | Insulin dependent diabetes mellitus with nephropathy |
| 91942 | C10E311 | Type I diabetes mellitus with multiple complications |
| 45276 | C10E312 | Insulin dependent diabetes mellitus with multiple complicat |
| 46963 | C108000 | Insulin-dependent diabetes mellitus with renal complications |
| 32556 | C107.12 | Diabetes with gangrene |
| 32403 | C107.11 | Diabetes mellitus with gangrene |
| 6509 | C108700 | Insulin dependent diabetes mellitus with retinopathy |
| 59253 | C10FG00 | Type 2 diabetes mellitus with arthropathy |
| 35288 | C10E800 | Type 1 diabetes mellitus - poor control |
| 68105 | C10EB00 | Type 1 diabetes mellitus with mononeuropathy |
| 34450 | C10FK00 | Hyperosmolar non-ketotic state in type 2 diabetes mellitus |
| 37648 | C109J11 | Insulin treated non-insulin dependent diabetes mellitus |
| 18264 | C109J12 | Insulin treated Type II diabetes mellitus |
| 50429 | C109100 | Non-insulin-dependent diabetes mellitus with ophthalm comps |
| 40023 | C102000 | Diabetes mellitus, juvenile type, with hyperosmolar coma |
| 68517 | C10J.00 | Insulin autoimmune syndrome |
| 18425 | C10FB00 | Type 2 diabetes mellitus with polyneuropathy |
| 39070 | C10EE00 | Type 1 diabetes mellitus with hypoglycaemic coma |
| 102946 | C10E012 | Insulin-dependent diabetes mellitus with renal complications |
| 109837 | C10E011 | Type I diabetes mellitus with renal complications |
| 48192 | C109E11 | Type II diabetes mellitus with diabetic cataract |
| 100347 | C10A500 | Malnutritn-relat diabetes melitus wth periph circul complctn |
| 22871 | C10EP00 | Type 1 diabetes mellitus with exudative maculopathy |
| 112402 | C107y00 | Other specified diabetes mellitus with periph circ comps |
| 57621 | C108D00 | Insulin dependent diabetes mellitus with nephropathy |
| 54212 | C109F00 | Non-insulin-dependent d m with peripheral angiopath |
| 16230 | C106.00 | Diabetes mellitus with neurological manifestation |
| 70316 | C109112 | Type 2 diabetes mellitus with ophthalmic complications |
| 59725 | C109111 | Type II diabetes mellitus with ophthalmic complications |
| 17545 | C108F11 | Type I diabetes mellitus with diabetic cataract |
| 66872 | C108D11 | Type I diabetes mellitus with nephropathy |
| 113197 | C108D12 | Type 1 diabetes mellitus with nephropathy |
| 57278 | C10F011 | Type II diabetes mellitus with renal complications |
| 64571 | C109C11 | Type II diabetes mellitus with nephropathy |
| 24836 | C109C12 | Type 2 diabetes mellitus with nephropathy |
| 63762 | C10z100 | Diabetes mellitus, adult onset, + unspecified complication |
| 85991 | C10FM11 | Type II diabetes mellitus with persistent microalbuminuria |
| 40962 | C109H00 | Non-insulin dependent d m with neuropathic arthropathy |
| 65616 | C108H00 | Insulin dependent diabetes mellitus with arthropathy |
| 55842 | C109200 | Non-insulin-dependent diabetes mellitus with neuro comps |
| 30294 | C10EL00 | Type 1 diabetes mellitus with persistent microalbuminuria |
| 65267 | C10F300 | Type 2 diabetes mellitus with multiple complications |
| 113863 | C10ED11 | Type I diabetes mellitus with nephropathy |
| 47650 | C10E300 | Type 1 diabetes mellitus with multiple complications |
| 35105 | C104100 | Diabetes mellitus, adult onset, with renal manifestation |
| 69676 | C10EA00 | Type 1 diabetes mellitus without complication |
| 33254 | C105.00 | Diabetes mellitus with ophthalmic manifestation |
| 15690 | C103.00 | Diabetes mellitus with ketoacidotic coma |
| 41389 | C105100 | Diabetes mellitus, adult onset, + ophthalmic manifestation |
| 62146 | C109300 | Non-insulin-dependent diabetes mellitus with multiple comps |
| 47377 | C105y00 | Other specified diabetes mellitus with ophthalmic complicatn |
| 65704 | C109412 | Type 2 diabetes mellitus with ulcer |
| 55075 | C109411 | Type II diabetes mellitus with ulcer |
| 41716 | C108C00 | Insulin dependent diabetes mellitus with polyneuropathy |
| 69124 | C107300 | IDDM with peripheral circulatory disorder |
| 49146 | C108211 | Type I diabetes mellitus with neurological complications |
| 1038 | C100011 | Insulin dependent diabetes mellitus |
| 111798 | C10FQ11 | Type II diabetes mellitus with exudative maculopathy |
| 53392 | C10F911 | Type II diabetes mellitus without complication |
| 99719 | C10EA12 | Insulin-dependent diabetes without complication |
| 94383 | C10N000 | Secondary diabetes mellitus without complication |
| 100770 | C10EF12 | Insulin dependent diabetes mellitus with diabetic cataract |
| 2475 | C104.11 | Diabetic nephropathy |
| 49276 | C108100 | Insulin-dependent diabetes mellitus with ophthalmic comps |
| 110611 | C10P111 | Type 2 diabetes mellitus in remission |
| 40682 | C10E900 | Type 1 diabetes mellitus maturity onset |
| 6791 | C108800 | Insulin dependent diabetes mellitus - poor control |
| 46917 | C10FD00 | Type 2 diabetes mellitus with hypoglycaemic coma |
| 43139 | C102100 | Diabetes mellitus, adult onset, with hyperosmolar coma |
| 12640 | C10FC00 | Type 2 diabetes mellitus with nephropathy |
| 18230 | C108J12 | Type 1 diabetes mellitus with neuropathic arthropathy |
| 60208 | C108J11 | Type I diabetes mellitus with neuropathic arthropathy |
| 44779 | C109E12 | Type 2 diabetes mellitus with diabetic cataract |
| 41049 | C108712 | Type 1 diabetes mellitus with retinopathy |
| 38161 | C108711 | Type I diabetes mellitus with retinopathy |
| 103902 | C10FG11 | Type II diabetes mellitus with arthropathy |
| 54899 | C109F11 | Type II diabetes mellitus with peripheral angiopathy |
| 60699 | C109F12 | Type 2 diabetes mellitus with peripheral angiopathy |
| 72702 | C10E812 | Insulin dependent diabetes mellitus - poor control |
| 105337 | C10E811 | Type I diabetes mellitus - poor control |
| 105784 | C109912 | Type 2 diabetes mellitus without complication |
| 109103 | C109911 | Type II diabetes mellitus without complication |
| 54600 | C10E412 | Unstable insulin dependent diabetes mellitus |
| 49949 | C10E411 | Unstable type I diabetes mellitus |
| 66675 | C10A000 | Malnutrition-related diabetes mellitus with coma |
| 30323 | C10EK00 | Type 1 diabetes mellitus with persistent proteinuria |
| 18777 | C10F000 | Type 2 diabetes mellitus with renal complications |
| 59365 | C109C00 | Non-insulin dependent diabetes mellitus with nephropathy |
| 12736 | C10F500 | Type 2 diabetes mellitus with gangrene |
| 93468 | C10EG00 | Type 1 diabetes mellitus with peripheral angiopathy |
| 66965 | C109H12 | Type 2 diabetes mellitus with neuropathic arthropathy |
| 47816 | C109H11 | Type II diabetes mellitus with neuropathic arthropathy |
| 69748 | C105000 | Diabetes mellitus, juvenile type, + ophthalmic manifestation |
| 67905 | C109211 | Type II diabetes mellitus with neurological complications |
| 45919 | C109212 | Type 2 diabetes mellitus with neurological complications |
| 102620 | C10EL11 | Type I diabetes mellitus with persistent microalbuminuria |
| 113975 | C108C11 | Type I diabetes mellitus with polyneuropathy |
| 43227 | C10F311 | Type II diabetes mellitus with multiple complications |
| 33807 | C107200 | Diabetes mellitus, adult with gangrene |
| 49655 | C10F611 | Type II diabetes mellitus with retinopathy |
| 106528 | C10FN11 | Type II diabetes mellitus with ketoacidosis |
| 52283 | C108200 | Insulin-dependent diabetes mellitus with neurological comps |
| 31310 | C108900 | Insulin dependent diabetes maturity onset |
| 44982 | C10FE00 | Type 2 diabetes mellitus with diabetic cataract |
| 4513 | C109.00 | Non-insulin dependent diabetes mellitus |
| 24490 | C100000 | Diabetes mellitus, juvenile type, no mention of complication |
| 47321 | C10F100 | Type 2 diabetes mellitus with ophthalmic complications |
| 1682 | C101.00 | Diabetes mellitus with ketoacidosis |
| 16502 | C104.00 | Diabetes mellitus with renal manifestation |
| 22573 | C106z00 | Diabetes mellitus NOS with neurological manifestation |
| 50972 | C100z00 | Diabetes mellitus NOS with no mention of complication |
| 64283 | C10zy00 | Other specified diabetes mellitus with unspecified comps |
| 68390 | C108512 | Type 1 diabetes mellitus with ulcer |
| 47315 | C10F711 | Type II diabetes mellitus - poor control |
| 61122 | C10H.00 | Diabetes mellitus induced by non-steroid drugs |
| 104323 | C10F511 | Type II diabetes mellitus with gangrene |
| 61829 | C108212 | Type 1 diabetes mellitus with neurological complications |
| 24458 | C109711 | Type II diabetes mellitus - poor control |
| 45913 | C109712 | Type 2 diabetes mellitus - poor control |
| 17858 | C108.12 | Type 1 diabetes mellitus |
| 24423 | C108.13 | Type I diabetes mellitus |
| 18505 | C108.11 | IDDM-Insulin dependent diabetes mellitus |
| 50813 | C109A11 | Type II diabetes mellitus with mononeuropathy |
| 102201 | C10FC11 | Type II diabetes mellitus with nephropathy |
| 114017 | C10J000 | Insulin autoimmune syndrome without complication |
| 39809 | C108J00 | Insulin dependent diab mell with neuropathic arthropathy |
| 113609 | C10FR11 | Type II diabetes mellitus with gastroparesis |
| 69278 | C109E00 | Non-insulin depend diabetes mellitus with diabetic cataract |
| 95343 | C10E711 | Type I diabetes mellitus with retinopathy |
| 93875 | C10E712 | Insulin dependent diabetes mellitus with retinopathy |
| 52303 | C109000 | Non-insulin-dependent diabetes mellitus with renal comps |
| 54008 | C10EJ00 | Type 1 diabetes mellitus with neuropathic arthropathy |
| 44440 | C108E00 | Insulin dependent diabetes mellitus with hypoglycaemic coma |
| 29979 | C109900 | Non-insulin-dependent diabetes mellitus without complication |
| 45467 | C109B00 | Non-insulin dependent diabetes mellitus with polyneuropathy |
| 49554 | C10EF00 | Type 1 diabetes mellitus with diabetic cataract |
| 68843 | C103100 | Diabetes mellitus, adult onset, with ketoacidotic coma |
| 18219 | C109.13 | Type II diabetes mellitus |
| 59288 | C103y00 | Other specified diabetes mellitus with coma |
| 98704 | C10E512 | Insulin dependent diabetes mellitus with ulcer |
| 109628 | C10P011 | Type 1 diabetes mellitus in remission |
| 56803 | C107400 | NIDDM with peripheral circulatory disorder |
| 40837 | C10EN00 | Type 1 diabetes mellitus with ketoacidotic coma |
| 60107 | C108411 | Unstable type I diabetes mellitus |
| 63371 | C10y100 | Diabetes mellitus, adult, + other specified manifestation |
| 53200 | C101000 | Diabetes mellitus, juvenile type, with ketoacidosis |
| 108724 | C10EQ11 | Type I diabetes mellitus with gastroparesis |
| 97894 | C10EP11 | Type I diabetes mellitus with exudative maculopathy |
| 52104 | C108300 | Insulin dependent diabetes mellitus with multiple complicatn |
| 46290 | C108y00 | Other specified diabetes mellitus with multiple comps |
| 46150 | C109512 | Type 2 diabetes mellitus with gangrene |
| 14803 | C100100 | Diabetes mellitus, adult onset, no mention of complication |
| 43921 | C10E400 | Unstable type 1 diabetes mellitus |
| 18496 | C10F600 | Type 2 diabetes mellitus with retinopathy |
| 21482 | C102.00 | Diabetes mellitus with hyperosmolar coma |
| 50225 | C109011 | Type II diabetes mellitus with renal complications |
| 32627 | C10FN00 | Type 2 diabetes mellitus with ketoacidosis |
| 51261 | C10E.12 | Insulin dependent diabetes mellitus |
| 38986 | C100.00 | Diabetes mellitus with no mention of complication |
| 35107 | C104z00 | Diabetes mellitus with nephropathy NOS |
| 72345 | C102z00 | Diabetes mellitus NOS with hyperosmolar coma |
| 63017 | C108911 | Type I diabetes mellitus maturity onset |
| 97446 | C108912 | Type 1 diabetes mellitus maturity onset |
| 93727 | C10FE11 | Type II diabetes mellitus with diabetic cataract |
| 5884 | C109.11 | NIDDM - Non-insulin dependent diabetes mellitus |
| 17859 | C109.12 | Type 2 diabetes mellitus |
| 100964 | C10F111 | Type II diabetes mellitus with ophthalmic complications |
| 98071 | C10E112 | Insulin-dependent diabetes mellitus with ophthalmic comps |
| 99311 | C10E111 | Type I diabetes mellitus with ophthalmic complications |
| 33343 | C10y.00 | Diabetes mellitus with other specified manifestation |
| 101735 | C10E212 | Insulin-dependent diabetes mellitus with neurological comps |
| 43785 | C109D00 | Non-insulin dependent diabetes mellitus with hypoglyca coma |
| 62209 | C10EM11 | Type I diabetes mellitus with ketoacidosis |
| 60796 | C10FL11 | Type II diabetes mellitus with persistent proteinuria |
| 8403 | C109700 | Non-insulin dependent diabetes mellitus - poor control |
| 1647 | C108.00 | Insulin dependent diabetes mellitus |
| 72320 | C109A00 | Non-insulin dependent diabetes mellitus with mononeuropathy |
| 109197 | C10FH11 | Type II diabetes mellitus with neuropathic arthropathy |
| 95636 | C10ER00 | Latent autoimmune diabetes mellitus in adult |
| 54856 | C101100 | Diabetes mellitus, adult onset, with ketoacidosis |
| 96506 | C10G000 | Secondary pancreatic diabetes mellitus without complication |
| 63690 | C10FR00 | Type 2 diabetes mellitus with gastroparesis |
| 42567 | C103000 | Diabetes mellitus, juvenile type, with ketoacidotic coma |
| 110400 | C108F12 | Type 1 diabetes mellitus with diabetic cataract |
| 18209 | C109012 | Type 2 diabetes mellitus with renal complications |
| 42729 | C108E11 | Type I diabetes mellitus with hypoglycaemic coma |
| 70766 | C108E12 | Type 1 diabetes mellitus with hypoglycaemic coma |
| 47409 | C109B11 | Type II diabetes mellitus with polyneuropathy |
| 109865 | C109B12 | Type 2 diabetes mellitus with polyneuropathy |
| 110997 | C10y000 | Diabetes mellitus, juvenile, + other specified manifestation |
| 67212 | C10H000 | DM induced by non-steroid drugs without complication |
| 70821 | C10yz00 | Diabetes mellitus NOS with other specified manifestation |
| 62107 | C109511 | Type II diabetes mellitus with gangrene |
| 91646 | C10F411 | Type II diabetes mellitus with ulcer |
| 97474 | C108412 | Unstable type 1 diabetes mellitus |
| 26108 | C10B000 | Steroid induced diabetes mellitus without complication |
| 26855 | C108400 | Unstable insulin dependent diabetes mellitus |
| 18387 | C10E700 | Type 1 diabetes mellitus with retinopathy |
| 64449 | C108z00 | Unspecified diabetes mellitus with multiple complications |
| 51697 | C10G.00 | Secondary pancreatic diabetes mellitus |
| 1549 | C10E.00 | Type 1 diabetes mellitus |
| 43453 | C10C.00 | Diabetes mellitus autosomal dominant |
| 25627 | C10F700 | Type 2 diabetes mellitus - poor control |
| 59991 | C10D.11 | Maturity onset diabetes in youth type 2 |
| 55239 | C10EQ00 | Type 1 diabetes mellitus with gastroparesis |
| 18143 | C109G11 | Type II diabetes mellitus with arthropathy |
| 108007 | C108311 | Type I diabetes mellitus with multiple complications |
| 67853 | C106000 | Diabetes mellitus, juvenile, + neurological manifestation |
| 102740 | C108112 | Type 1 diabetes mellitus with ophthalmic complications |
| 14889 | C100111 | Maturity onset diabetes |
| 506 | C100112 | Non-insulin dependent diabetes mellitus |
| 37957 | C10K.00 | Type A insulin resistance |
| 35385 | C10FH00 | Type 2 diabetes mellitus with neuropathic arthropathy |
| 114401 | C108612 | Type 1 diabetes mellitus with gangrene |
| 104639 | C10FF11 | Type II diabetes mellitus with peripheral angiopathy |
| 22884 | C10F.11 | Type II diabetes mellitus |
| 49869 | C109G12 | Type 2 diabetes mellitus with arthropathy |
| 96235 | C10E911 | Type I diabetes mellitus maturity onset |
| 113495 | C109311 | Type II diabetes mellitus with multiple complications |
| 35397 | C1A..00 | Insulin resistance |
| 12724 | C1A0.00 | Metabolic syndrome |
| 52212 | Cyu2.00 | [X]Diabetes mellitus |
| 100292 | Cyu2300 | [X]Unspecified diabetes mellitus with renal complications |
| 41686 | Cyu2000 | [X]Other specified diabetes mellitus |
| 37315 | F3y0.00 | Diabetic mononeuropathy |
| 44033 | F345000 | Diabetic mononeuritis multiplex |
| 17247 | F35z000 | Diabetic mononeuritis NOS |
| 5002 | F372.11 | Diabetic polyneuropathy |
| 2342 | F372.12 | Diabetic neuropathy |
| 31790 | F372.00 | Polyneuropathy in diabetes |
| 24571 | F372200 | Asymptomatic diabetic neuropathy |
| 48078 | F372000 | Acute painful diabetic neuropathy |
| 35785 | F372100 | Chronic painful diabetic neuropathy |
| 47584 | F420500 | Advanced diabetic retinal disease |
| 1323 | F420.00 | Diabetic retinopathy |
| 3837 | F420400 | Diabetic maculopathy |
| 10099 | F420300 | Advanced diabetic maculopathy |
| 10755 | F420600 | Non proliferative diabetic retinopathy |
| 7069 | F420000 | Background diabetic retinopathy |
| 30477 | F420700 | High risk proliferative diabetic retinopathy |
| 11626 | F420z00 | Diabetic retinopathy NOS |
| 3286 | F420100 | Proliferative diabetic retinopathy |
| 65463 | F420800 | High risk non proliferative diabetic retinopathy |
| 2986 | F420200 | Preproliferative diabetic retinopathy |
| 10659 | F464000 | Diabetic cataract |
| 11663 | M271100 | Neuropathic diabetic ulcer - foot |
| 9881 | M271200 | Mixed diabetic ulcer - foot |
| 24327 | M271000 | Ischaemic ulcer diabetic foot |
| 25446 | R10D.00 | [D]Elevated blood glucose level |
| 94904 | R10D100 | [D]Stress induced hyperglycaemia |
| 10791 | R10D000 | [D]Impaired fasting glycaemia |
| 31161 | R10D011 | [D]Impaired fasting glucose |
| 3505 | C313500 | Glucose intolerance |
| 11848 | C314.11 | Renal diabetes |
| 101801 | 66At100 | Type II diabetic dietary review |
| 43951 | 66AK.00 | Diabetic - cooperative patient |
| 8836 | 66AR.00 | Diabetes management plan given |
| 18167 | 66AT.00 | Annual diabetic blood test |
| 28769 | 66AV.00 | Diabetic on insulin and oral treatment |
| 13281 | 66A7.00 | Frequency of hypo. attacks |
| 66274 | 66Ah.00 | Insulin needles changed for each injection |
| 69152 | 66Aj.00 | Insulin needles changed less than once a day |
| 61470 | 66Al.00 | Diabetic monitoring - higher risk albumin excretion |
| 37035 | 66Ae.00 | HbA1c target |
| 90301 | 66Ag.00 | Insulin needles changed daily |
| 22959 | 66AJ000 | Chronic hyperglycaemia |
| 12675 | 66AQ.00 | Diabetes: shared care programme |
| 55123 | 66AO.00 | Date diabetic treatment stopp. |
| 102704 | 66At000 | Type I diabetic dietary review |
| 46577 | 66AX.00 | Diabetes: shared care in pregnancy - diabetol and obstet |
| 13067 | 66AZ.00 | Diabetic monitoring NOS |
| 13193 | 66AC.00 | Blood sugar charts |
| 13291 | 66AE.00 | Feet examination |
| 53238 | 66AG.00 | Diabetic drug side effects |
| 6125 | 66AS.00 | Diabetic annual review |
| 12307 | 66AU.00 | Diabetes care by hospital only |
| 50175 | 66AW.00 | Diabetic foot risk assessment |
| 101190 | 66AQ100 | Declined consent for diabetes year of care programme |
| 31752 | 66A7000 | Frequency of hospital treated hypoglycaemia |
| 85660 | 66An.00 | Diabetes type 1 review |
| 13069 | 66A8.00 | Has seen dietician - diabetes |
| 28873 | 66Ai.00 | Diabetic 6 month review |
| 66475 | 66Ak.00 | Diabetic monitoring - lower risk albumin excretion |
| 100791 | 66Ar.00 | Insulin treatment stopped |
| 101177 | 66At.00 | Diabetic dietary review |
| 104453 | 66At011 | Type 1 diabetic dietary review |
| 108218 | 66AJ400 | Hypoglycaemic warning absent |
| 13070 | 66A1.00 | Initial diabetic assessment |
| 40363 | 66A7100 | Frequency of GP or paramedic treated hypoglycaemia |
| 26604 | 66AY.00 | Diabetic diet - good compliance |
| 83485 | 66Am.00 | Insulin dose changed |
| 107331 | 66AH100 | Conversion to insulin in secondary care |
| 16490 | 66AH.00 | Diabetic treatment changed |
| 3550 | 66A..00 | Diabetic monitoring |
| 608 | 66A2.00 | Follow-up diabetic assessment |
| 1684 | 66A4.00 | Diabetic on oral treatment |
| 38078 | 66A9.00 | Understands diet - diabetes |
| 101728 | 66As.00 | Diabetic on subcutaneous treatment |
| 102434 | 66Au.00 | Diabetic erectile dysfunction review |
| 37625 | 66AJ300 | Recurrent severe hypos |
| 96010 | 66Ap.00 | Insulin treatment initiated |
| 17886 | 66AM.00 | Diabetic - follow-up default |
| 102490 | 66Av.00 | Diabetic assessment of erectile dysfunction |
| 22823 | 66Ab.00 | Diabetic foot examination |
| 103847 | 66Ax.00 | Checking accuracy of blood glucose meter |
| 22023 | 66AJz00 | Diabetic - poor control NOS |
| 101208 | 66Ae000 | HbA1c target level - IFCC standardised |
| 11047 | 66AH000 | Conversion to insulin |
| 107464 | 66AS000 | Diabetes Year of Care annual review |
| 9013 | 66AJ.11 | Unstable diabetes |
| 111211 | 66Ad000 |  |
| 21420 | 66AJ200 | Loss of hypoglycaemic warning |
| 107508 | 66AH200 | Conversion to insulin by diabetes specialist nurse |
| 2478 | 66AJ100 | Brittle diabetes |
| 13071 | 66AI.00 | Diabetic - good control |
| 25636 | 66Aa.00 | Diabetic diet - poor compliance |
| 7563 | 66A3.00 | Diabetic on diet only |
| 8842 | 66A5.00 | Diabetic on insulin |
| 2378 | 66AJ.00 | Diabetic - poor control |
| 17869 | 66AL.00 | Diabetic-uncooperative patient |
| 12506 | 66AP.00 | Diabetes: practice programme |
| 109700 | 66AH300 | Conversion to non-insulin injectable medication |
| 13068 | 66A6.00 | Last hypo. attack |
| 102611 | 66At111 | Type 2 diabetic dietary review |
| 18583 | 66Ad.00 | Hypoglycaemic attack requiring 3rd party assistance |
| 32619 | 66Af.00 | Patient diabetes education review |
| 100533 | 66AQ000 | Unsuitable for diabetes year of care programme |
| 95994 | 66Aq.00 | Diabetic foot screen |
| 83532 | 66Ao.00 | Diabetes type 2 review |
| 20696 | 66AA.11 | Injection sites - diabetic |
| 102549 | 66Aw.00 | Insulin dose |
| 10977 | 66Ac.00 | Diabetic peripheral neuropathy screening |
| 26510 | 66AB.00 | Urine sugar charts |
| 29041 | 66AN.00 | Date diabetic treatment start |
| 13196 | 66AD.00 | Fundoscopy - diabetic check |
| 6430 | 9NM0.00 | Attending diabetes clinic |
| 107739 | 679L211 | Advice about diabetes and driving |
| 106358 | 679L100 | Hypoglycaemia education |
| 13057 | 679L.00 | Health education - diabetes |
| 107361 | 679L200 | Education about diabetes and driving |
| 108890 | 679L300 | Diabetic foot care education |
| 100436 | 679L000 | Education in self management of diabetes |
| 18056 | 2G5C.00 | Foot abnormality - diabetes related |
| 17067 | F171100 | Autonomic neuropathy due to diabetes |
| 2471 | K01x100 | Nephrotic syndrome in diabetes mellitus |
| 11930 | 9NN9.00 | Under care of diabetes specialist nurse |
| 38103 | 9N0m.00 | Seen in diabetic nurse consultant clinic |
| 16881 | ZV65312 | [V]Dietary counselling in diabetes mellitus |
| 32739 | 9N0n.00 | Seen in community diabetes specialist clinic |
| 7045 | 14F4.00 | H/O: Admission in last year for diabetes foot problem |
| 113796 | 9Na5200 | Joint consultation GP & community diabetes specialist nurse |
| 111347 | 9Na5100 | Joint consultn pracse nurse & comm diabetes specialist nurse |
| 13074 | 13B1.00 | Diabetic diet |
| 110393 | 13B1000 | Diabetic carbohydrate counting diet |
| 17313 | F440700 | Diabetic iritis |
| 103798 | 9b92000 | Diabetic medicine |
| 39420 | F381300 | Myasthenic syndrome due to diabetic amyotrophy |
| 2340 | F381311 | Diabetic amyotrophy |
| 9974 | 9N1v.00 | Seen in diabetic eye clinic |
| 7328 | M037200 | Cellulitis in diabetic foot |
| 10824 | 9N1i.00 | Seen in diabetic foot clinic |
| 13078 | 13AC.00 | Diabetic weight reducing diet |
| 34152 | G73y000 | Diabetic peripheral angiopathy |
| 12507 | 9N2i.00 | Seen by diabetic liaison nurse |
| 53634 | R054200 | [D]Gangrene of toe in diabetic |
| 13103 | 2BBS.00 | O/E - left eye preproliferative diabetic retinopathy |
| 52041 | 2BBl.00 | O/E - left eye stable treated prolif diabetic retinopathy |
| 11433 | 2BBP.00 | O/E - right eye background diabetic retinopathy |
| 22967 | 2BBF.00 | Retinal abnormality - diabetes related |
| 13099 | 2BBR.00 | O/E - right eye preproliferative diabetic retinopathy |
| 9835 | 2BBL.00 | O/E - diabetic maculopathy present both eyes |
| 13102 | 2BBW.00 | O/E - right eye diabetic maculopathy |
| 13108 | 2BBX.00 | O/E - left eye diabetic maculopathy |
| 13097 | 2BBT.00 | O/E - right eye proliferative diabetic retinopathy |
| 13101 | 2BBV.00 | O/E - left eye proliferative diabetic retinopathy |
| 11129 | 2BBQ.00 | O/E - left eye background diabetic retinopathy |
| 52630 | 2BBo.00 | O/E - sight threatening diabetic retinopathy |
| 101881 | 2BBr.00 | Impaired vision due to diabetic retinopathy |
| 11094 | 9NND.00 | Under care of diabetic foot screener |
| 31053 | R054300 | [D]Widespread diabetic foot gangrene |
| 57389 | 93C4.00 | Patient consent given for addition to diabetic register |
| 105434 | C11y400 | Impaired glucose regulation |
| 10921 | C11y200 | Impaired glucode tolerance |
| 2664 | L180900 | Gestational diabetes mellitus |
| 8446 | L180811 | Gestational diabetes mellitus |
| 9958 | 42W..00 | Hb. A1C - diabetic control |
| 10278 | L180800 | Diabetes mellitus arising in pregnancy |
| 10642 | ZC2C800 | Dietary advice for diabetes mellitus |
| 11359 | L180.00 | Diabetes mellitus during pregnancy/childbirth/puerperium |
| 12682 | 679R.00 | Patient offered diabetes structured education programme |
| 14049 | 42WZ.00 | Hb. A1C - diabetic control NOS |
| 14050 | 42c..00 | HbA1 - diabetic control |
| 17095 | 2G5A.00 | O/E - Right diabetic foot at risk |
| 18142 | N030000 | Diabetic cheiroarthropathy |
| 18311 | 68A7.00 | Diabetic retinopathy screening |
| 21689 | 13AB.00 | Diabetic lipid lowering diet |
| 23479 | C350011 | Bronzed diabetes |
| 25041 | ZC2CA00 | Dietary advice for type II diabetes |
| 26664 | 2G5B.00 | O/E - Left diabetic foot at risk |
| 26666 | 2G5E.00 | O/E - Right diabetic foot at low risk |
| 26667 | 2G5I.00 | O/E - Left diabetic foot at low risk |
| 27891 | N030100 | Diabetic Charcot arthropathy |
| 27921 | 2G51000 | Foot abnormality - diabetes related |
| 31156 | 2G5J.00 | O/E - Left diabetic foot at moderate risk |
| 31157 | 2G5F.00 | O/E - Right diabetic foot at moderate risk |
| 31171 | 2G5G.00 | O/E - Right diabetic foot at high risk |
| 31172 | 2G5K.00 | O/E - Left diabetic foot at high risk |
| 32193 | C11y000 | Steroid induced diabetes |
| 32359 | ZRbH.00 | Perceived control of insulin-dependent diabetes |
| 34639 | L180100 | Diabetes mellitus during pregnancy - baby delivered |
| 35116 | 2G5L.00 | O/E - Left diabetic foot - ulcerated |
| 35316 | 2G5H.00 | O/E - Right diabetic foot - ulcerated |
| 45499 | K01x111 | Kimmelstiel - Wilson disease |
| 47328 | 2BBk.00 | O/E - right eye stable treated prolif diabetic retinopathy |
| 49559 | L180300 | Diabetes mellitus during pregnancy - baby not yet delivered |
| 49640 | 2G5W.00 | O/E - left chronic diabetic foot ulcer |
| 49884 | 6761 | Diabetic pre-pregnancy counselling |
| 50609 | L180600 | Pre-existing diabetes mellitus, non-insulin-dependent |
| 50960 | L180500 | Pre-existing diabetes mellitus, insulin-dependent |
| 55431 | L180X00 | Pre-existing diabetes mellitus, unspecified |
| 57333 | N030011 | Diabetic cheiropathy |
| 62384 | 2G5V.00 | O/E - right chronic diabetic foot ulcer |
| 64384 | L180z00 | Diabetes mellitus in pregnancy/childbirth/puerperium NOS |
| 67635 | L180000 | Diabetes mellitus - unspec whether in pregnancy/puerperium |
| 69043 | ZC2C900 | Dietary advice for type I diabetes |
| 72385 | 9Ol4.00 | Mental health monitoring telephone invite |
| 96823 | L180400 | Diabetes mellitus in pueperium - baby previously delivered |
| 99628 | Kyu0300 | [X]Glomerular disorders in diabetes mellitus |

**Supplementary Table 2: Medication Codes**

| **PRODCODE** | **PRODUCTNAME** |
| --- | --- |
| 1592 | Actrapid penfill 100 100iu/ml Penfill (Novo Nordisk Ltd) |
| 13622 | Hypurin porcine neutral 100unit/ml Injection (C P Pharmaceuticals Ltd) |
| 18224 | Humalog 100units/ml solution for injection 10ml vials (Eli Lilly and Company Ltd) |
| 22945 | Insuman rapid 100iu/ml Injection (Aventis Pharma) |
| 29953 | Apidra 100units/ml solution for injection 3ml OptiClik cartridges (Sanofi) |
| 16142 | Insulin aspart 100units/ml solution for injection 3ml cartridges |
| 1588 | Actrapid 100iu/ml Injection (Novo Nordisk Ltd) |
| 76991 | Humalog 100units/ml solution for injection 10ml vials (Waymade Healthcare Plc) |
| 21583 | Apidra 100units/ml solution for injection 3ml pre-filled OptiSet pen (Sanofi) |
| 29567 | Insulin aspart 100units/ml solution for injection 10ml vials |
| 53251 | NovoRapid Penfill 100units/ml solution for injection 3ml cartridges (DE Pharmaceuticals) |
| 18592 | Insulin soluble bovine 100units/ml solution for injection 10ml vials |
| 7318 | Humalog 100units/ml solution for injection 3ml cartridges (Eli Lilly and Company Ltd) |
| 57529 | Humalog 100units/ml solution for injection 10ml vials (Dowelhurst Ltd) |
| 28101 | Insulin glulisine 100units/ml solution for injection 10ml vials |
| 27396 | Insulin soluble porcine 100units/ml solution for injection 10ml vials |
| 26060 | Insulin lispro 100units/ml solution for injection 10ml vials |
| 57564 | Humalog KwikPen 100units/ml solution for injection 3ml pre-filled pen (Waymade Healthcare Plc) |
| 76660 | Insulin lispro Sanofi 100units/ml solution for injection 3ml cartridges (Sanofi) |
| 55603 | Humalog KwikPen 100units/ml solution for injection 3ml pre-filled pen (DE Pharmaceuticals) |
| 77012 | Humalog 100units/ml solution for injection 10ml vials (DE Pharmaceuticals) |
| 5892 | NovoRapid FlexPen 100units/ml solution for injection 3ml pre-filled pen (Novo Nordisk Ltd) |
| 7349 | Actrapid 100units/ml solution for injection 10ml vials (Novo Nordisk Ltd) |
| 51743 | NovoRapid Penfill 100units/ml solution for injection 3ml cartridges (Sigma Pharmaceuticals Plc) |
| 14938 | Insulin soluble bovine cartridge 100unit/ml Solution for injection |
| 4706 | Velosulin 100units/ml solution for injection 10ml vials (Novo Nordisk Ltd) |
| 11337 | NovoRapid Novolet 100units/ml solution for injection (Novo Nordisk Ltd) |
| 53118 | NovoRapid FlexPen 100units/ml solution for injection 3ml pre-filled pen (Mawdsley-Brooks & Company Ltd) |
| 14313 | Insulin lispro 100units/ml solution for injection 3ml cartridges |
| 77805 | Insulin lispro 200units/ml solution for injection 3ml pre-filled disposable devices |
| 14339 | Hypurin Bovine Neutral 100units/ml solution for injection 10ml vials (Wockhardt UK Ltd) |
| 23231 | Hypurin Bovine Neutral 100units/ml solution for injection 3ml cartridges (Wockhardt UK Ltd) |
| 63679 | Hypurin soluble 100iu/ml Injection (C P Pharmaceuticals Ltd) |
| 74824 | Actrapid 100units/ml solution for injection 10ml vials (Waymade Healthcare Plc) |
| 26098 | Hypurin Porcine Neutral 100units/ml solution for injection 10ml vials (Wockhardt UK Ltd) |
| 28442 | Insulin glulisine 100unit/ml Solution for injection |
| 62180 | Insulin aspart 100units/ml solution for injection 1.6ml cartridges |
| 6209 | NovoRapid 100units/ml solution for injection 10ml vials (Novo Nordisk Ltd) |
| 75329 | Insulin lispro Sanofi 100units/ml solution for injection 3ml pre-filled pen (Sanofi) |
| 6447 | Insulin aspart human pyr 100 iu/ml Injection |
| 74389 | Humalog KwikPen 100units/ml solution for injection 3ml pre-filled pen (Sigma Pharmaceuticals Plc) |
| 14930 | Hypurin Porcine Neutral 100units/ml solution for injection 3ml cartridges (Wockhardt UK Ltd) |
| 25479 | Insulin soluble porcine 100units/ml solution for injection 3ml cartridges |
| 59533 | NovoRapid FlexPen 100units/ml solution for injection 3ml pre-filled pen (Sigma Pharmaceuticals Plc) |
| 41959 | Penject 100unit/ml Injection device (Hypoguard Ltd) |
| 74479 | Insulin lispro 100units/ml solution for injection 3ml pre-filled pen (Sanofi) |
| 24846 | Pur-in neutral 100unit/ml Injection (C P Pharmaceuticals Ltd) |
| 14362 | Insulin lispro 100units/ml solution for injection 3ml pre-filled disposable devices |
| 1842 | Pork velosulin 100unit/ml Injection (Novo Nordisk Ltd) |
| 49108 | NovoRapid Penfill 100units/ml solution for injection 3ml cartridges (Necessity Supplies Ltd) |
| 74179 | NovoRapid FlexPen 100units/ml solution for injection 3ml pre-filled pen (Waymade Healthcare Plc) |
| 14345 | Apidra 100units/ml solution for injection 3ml cartridges (Sanofi) |
| 12654 | Insulin soluble human prb 100unit/ml Injection |
| 17336 | Novopen 100unit/ml Injection device (Novo Nordisk Ltd) |
| 76164 | Humalog 100units/ml solution for injection 3ml cartridges (Sigma Pharmaceuticals Plc) |
| 21590 | Insulin glulisine 100units/ml solution for injection 3ml pre-filled disposable devices |
| 61845 | NovoRapid PumpCart 100units/ml solution for injection 1.6ml cartridges (Novo Nordisk Ltd) |
| 36513 | Velosulin cartridge 100unit/ml Injection (Novo Nordisk Ltd) |
| 16129 | Insulin soluble human 100units/ml solution for injection 3ml cartridges |
| 46666 | NovoRapid FlexTouch 100units/ml solution for injection 3ml pre-filled pen (Novo Nordisk Ltd) |
| 19877 | Insulin aspart 100units/ml solution for injection 3ml pre-filled disposable devices |
| 67313 | NovoRapid 100units/ml solution for injection 10ml vials (Sigma Pharmaceuticals Plc) |
| 38986 | Humalog KwikPen 100units/ml solution for injection 3ml pre-filled pen (Eli Lilly and Company Ltd) |
| 70055 | Fiasp Penfill 100units/ml solution for injection 3ml cartridges (Novo Nordisk Ltd) |
| 1840 | Humulin s 100unit/ml Injection (Eli Lilly and Company Ltd) |
| 22983 | Insuman Rapid 100units/ml solution for injection 3ml cartridges (Sanofi) |
| 63464 | Humalog KwikPen 200units/ml solution for injection 3ml pre-filled pen (Eli Lilly and Company Ltd) |
| 15710 | Insulin soluble human emp 100unit/ml Injection |
| 76533 | Insulin lispro Sanofi 100units/ml solution for injection 10ml vials (Sanofi) |
| 74392 | Apidra 100units/ml solution for injection 3ml pre-filled SoloStar pen (Lexon (UK) Ltd) |
| 5021 | NovoRapid Penfill 100units/ml solution for injection 3ml cartridges (Novo Nordisk Ltd) |
| 21235 | Humulin S 100units/ml solution for injection 10ml vials (Eli Lilly and Company Ltd) |
| 26621 | Insulin soluble human crb 100iu/ml Injection |
| 24593 | Neutral insulin bovine 100unit/ml Injection |
| 30209 | Actrapid mc 100unit/ml Injection (Arun Products Ltd) |
| 12297 | Hypurin bovine neutral 100unit/ml Injection (C P Pharmaceuticals Ltd) |
| 12638 | Insulin soluble human pyr 100unit/ml Injection |
| 73282 | Humalog Junior KwikPen 100units/ml solution for injection 3ml pre-filled pen (Eli Lilly and Company Ltd) |
| 36920 | Apidra 100units/ml solution for injection 3ml pre-filled SoloStar pen (Sanofi) |
| 23993 | Insuman Rapid 100units/ml solution for injection 3ml pre-filled OptiSet pen (Sanofi) |
| 10264 | Humalog Pen 100units/ml solution for injection 3ml pre-filled pen (Eli Lilly and Company Ltd) |
| 14944 | Humulin S 100units/ml solution for injection 3ml cartridges (Eli Lilly and Company Ltd) |
| 9521 | Pork Actrapid 100units/ml solution for injection 10ml vials (Novo Nordisk Ltd) |
| 10572 | Insulin soluble bovine 100unit/ml Injection |
| 67231 | NovoRapid FlexPen 100units/ml solution for injection 3ml pre-filled pen (Dowelhurst Ltd) |
| 27402 | Insulin soluble human 100units/ml solution for injection 10ml vials |
| 56502 | Actrapid Penfill 100units/ml solution for injection 3ml cartridges (Novo Nordisk Ltd) |
| 19491 | Apidra 100units/ml solution for injection 10ml vials (Sanofi) |
| 77059 | Pork Actrapid 100units/ml solution for injection 10ml vials (Waymade Healthcare Plc) |
| 14299 | Insulin glulisine 100units/ml solution for injection 3ml cartridges |
| 69823 | Fiasp FlexTouch 100units/ml solution for injection 3ml pre-filled pen (Novo Nordisk Ltd) |
| 47360 | Neutral insulin 100unit/ml Injection (Celltech Pharma Europe Ltd) |
| 76975 | Humalog 100units/ml solution for injection 10ml vials (Sigma Pharmaceuticals Plc) |
| 69715 | Fiasp 100units/ml solution for injection 10ml vials (Novo Nordisk Ltd) |
| 43950 | Humulin I KwikPen 100units/ml suspension for injection 3ml pre-filled pen (Eli Lilly and Company Ltd) |
| 4163 | Rapitard MC 100unit/ml Injection (Novo Nordisk Ltd) |
| 28588 | Hypurin Bovine Isophane 100units/ml suspension for injection 3ml cartridges (Wockhardt UK Ltd) |
| 7400 | Insulin glargine 100units/ml solution for injection 3ml pre-filled disposable devices |
| 5953 | Insulin glargine 100iu/ml Injection |
| 55687 | Insulin degludec 100units/ml solution for injection 3ml pre-filled disposable devices |
| 10207 | Insulin isophane human 100units/ml suspension for injection 3ml cartridges |
| 14928 | Insulatard 100units/ml suspension for injection 10ml vials (Novo Nordisk Ltd) |
| 10225 | Lantus 100units/ml solution for injection 3ml OptiClik cartridges (Sanofi) |
| 30236 | Isophane insulin 100iu/ml Injection |
| 10910 | Humaject m2 100iu/ml M2 pen (Eli Lilly and Company Ltd) |
| 71351 | Lantus 100units/ml solution for injection 3ml cartridges (Waymade Healthcare Plc) |
| 56691 | Insulin degludec 200units/ml solution for injection 3ml pre-filled disposable devices |
| 74970 | Pur-n Isophane 100unit/ml Injection (C P Pharmaceuticals Ltd) |
| 36066 | Insulin isophane bovine 100units/ml suspension for injection 3ml cartridges |
| 8118 | Humaject i 100iu/ml Pen (Eli Lilly and Company Ltd) |
| 8203 | Penmix 50/50 100iu/ml Penfill (Novo Nordisk Ltd) |
| 71428 | Insulatard InnoLet 100units/ml suspension for injection 3ml pre-filled pen (Waymade Healthcare Plc) |
| 71430 | Humalog Mix25 Pen 100units/ml suspension for injection 3ml pre-filled pen (Waymade Healthcare Plc) |
| 6958 | Levemir FlexPen 100units/ml solution for injection 3ml pre-filled pen (Novo Nordisk Ltd) |
| 44251 | Insulin zinc suspension mixed porcine 100unit/ml Injection |
| 35468 | Insuman Basal 100units/ml suspension for injection 5ml vials (Sanofi) |
| 10067 | Insulin biphasic aspart human pyr 30:70; 100 units/ml Injection |
| 69583 | Humalog Mix25 100units/ml suspension for injection 3ml cartridges (Waymade Healthcare Plc) |
| 4199 | Humulin m1 100unit/ml M1 injection (Eli Lilly and Company Ltd) |
| 9376 | Insulin zinc suspension crystalline human pyr 100unit/ml long acting Injection |
| 9737 | Insulatard innolet 100iu/ml Injection (Novo Nordisk Ltd) |
| 21554 | Insuman comb 50 100iu/ml Injection (Aventis Pharma) |
| 11107 | Humulin m4 100unit/ml M4 injection (Eli Lilly and Company Ltd) |
| 10887 | Penmix 40/60 100iu/ml Penfill (Novo Nordisk Ltd) |
| 16682 | Tempulin 100unit/ml Injection (Knoll Ltd) |
| 3551 | Mixtard 20 penfill 100 100iu/ml Penfill (Novo Nordisk Ltd) |
| 55462 | Tresiba FlexTouch 100units/ml solution for injection 3ml pre-filled pen (Novo Nordisk Ltd) |
| 5250 | Insulin biphasic lispro human prb 25:75; 100 units/ml Injection |
| 22155 | Humaject m5 100iu/ml M5 pen (Eli Lilly and Company Ltd) |
| 11056 | Insulin biphasic isophane human pyr 30:70; 100 units/ml Injection |
| 14290 | Insulatard Penfill 100units/ml suspension for injection 3ml cartridges (Novo Nordisk Ltd) |
| 76512 | Insulin biphasic isophane human emp 30:70; 100 units/ml Injection |
| 71395 | Humalog Mix25 100units/ml suspension for injection 3ml cartridges (Sigma Pharmaceuticals Plc) |
| 57622 | Humalog Mix50 KwikPen 100units/ml suspension for injection 3ml pre-filled pen (Waymade Healthcare Plc) |
| 17809 | Humaject m4 100iu/ml M4 pen (Eli Lilly and Company Ltd) |
| 67279 | Pork Insulatard 100units/ml suspension for injection 10ml vials (Waymade Healthcare Plc) |
| 1649 | Human actraphane 100iu/ml Injection (Novo Nordisk Ltd) |
| 22058 | Pur-in mix 15/85 Injection (C P Pharmaceuticals Ltd) |
| 55910 | Tresiba Penfill 100units/ml solution for injection 3ml cartridges (Novo Nordisk Ltd) |
| 14649 | Insulin biphasic isophane human pyr 10:90; 100 units/ml Injection |
| 74862 | Lantus 100units/ml solution for injection 3ml pre-filled OptiSet pen (Mawdsley-Brooks & Company Ltd) |
| 2929 | Mixtard 30 100iu/ml GE injection (Novo Nordisk Ltd) |
| 55618 | Levemir FlexPen 100units/ml solution for injection 3ml pre-filled pen (Waymade Healthcare Plc) |
| 55234 | Tresiba FlexTouch 200units/ml solution for injection 3ml pre-filled pen (Novo Nordisk Ltd) |
| 27461 | Insuman Basal 100units/ml suspension for injection 3ml cartridges (Sanofi) |
| 17731 | Penmix 50/50 100iu/ml Injection (Novo Nordisk Ltd) |
| 33966 | Insulatard 100unit/ml Injection (Novo Nordisk Ltd) |
| 35260 | Levemir InnoLet 100units/ml solution for injection 3ml pre-filled pen (Novo Nordisk Ltd) |
| 6965 | Levemir Penfill 100units/ml solution for injection 3ml cartridges (Novo Nordisk Ltd) |
| 66335 | Insulin biphasic isophane porcine 50:50; 100 units/ml Injection |
| 6061 | Novomix 30 30/70 100units/ml Injection (Novo Nordisk Ltd) |
| 14301 | Insulin detemir 100units/ml solution for injection 3ml cartridges |
| 2454 | Mixtard 30 penfill 100 100iu/ml Penfill (Novo Nordisk Ltd) |
| 64354 | Toujeo 300units/ml solution for injection 1.5ml pre-filled SoloStar pen (Sanofi) |
| 1806 | Penmix 30/70 100iu/ml Penfill (Novo Nordisk Ltd) |
| 14918 | Humulin I 100units/ml suspension for injection 10ml vials (Eli Lilly and Company Ltd) |
| 13729 | Insulin isophane human emp 100unit/ml Injection |
| 8322 | Insulin zinc suspension mixed human pyr 100unit/ml Injection |
| 1843 | Pork Insulatard 100units/ml suspension for injection 10ml vials (Novo Nordisk Ltd) |
| 16700 | Insulin zinc mixed bovine vial 100unit/ml Sterile suspension injection |
| 36853 | Lantus 100units/ml solution for injection 3ml pre-filled SoloStar pen (Sanofi) |
| 26403 | Pur-in mix 25/75 Injection (C P Pharmaceuticals Ltd) |
| 10484 | Penmix 20/80 Penfill (Novo Nordisk Ltd) |
| 52522 | Humalog Mix50 KwikPen 100units/ml suspension for injection 3ml pre-filled pen (DE Pharmaceuticals) |
| 18593 | Humalog Mix50 100units/ml suspension for injection 3ml cartridges (Eli Lilly and Company Ltd) |
| 14505 | Insulin protamine zinc bovine 100units/ml suspension for injection 10ml vials |
| 7266 | Lantus 100units/ml solution for injection 3ml cartridges (Sanofi) |
| 55907 | Insulin degludec 100units/ml solution for injection 3ml cartridges |
| 3550 | Mixtard 40 penfill 100 100iu/ml Penfill (Novo Nordisk Ltd) |
| 34031 | Monotard mc 100unit/ml Injection (Novo Nordisk Ltd) |
| 18590 | Insulin isophane bovine 100units/ml suspension for injection 10ml vials |
| 35701 | Insulin lispro biphasic 50/50 100units/ml suspension for injection 3ml pre-filled disposable devices |
| 10001 | Humalog Mix50 Pen 100units/ml suspension for injection 3ml pre-filled pen (Eli Lilly and Company Ltd) |
| 7350 | Insulin isophane porcine 100units/ml suspension for injection 10ml vials |
| 4760 | Humulin i 100unit/ml Injection (Eli Lilly and Company Ltd) |
| 4198 | Humulin m3 100unit/ml M3 injection (Eli Lilly and Company Ltd) |
| 1593 | Insulatard penfill 100 100iu/ml Penfill (Novo Nordisk Ltd) |
| 26498 | Insulin zinc suspension mixed bovine and porcine 100unit/ml Injection |
| 15199 | Insuman comb 25 100iu/ml Injection (Aventis Pharma) |
| 68031 | Humalog Mix25 KwikPen 100units/ml suspension for injection 3ml pre-filled pen (Sigma Pharmaceuticals Plc) |
| 10259 | Insulin glargine 100units/ml solution for injection 10ml vials |
| 14644 | Insulin biphasic isophane human prb 20:80; 100 units/ml Injection |
| 67230 | Lantus 100units/ml solution for injection 3ml pre-filled SoloStar pen (Waymade Healthcare Plc) |
| 21110 | Insulin biphasic isophane human prb 50:50; 100 units/ml Injection |
| 23992 | Insuman Basal 100units/ml suspension for injection 3ml pre-filled OptiSet pen (Sanofi) |
| 43953 | Insulin lispro biphasic 25/75 100units/ml suspension for injection 10ml vials |
| 39086 | Humalog Mix50 KwikPen 100units/ml suspension for injection 3ml pre-filled pen (Eli Lilly and Company Ltd) |
| 71137 | Hypurin Bovine Isophane 100units/ml suspension for injection 10ml vials (Waymade Healthcare Plc) |
| 62899 | Xultophy 100units/ml / 3.6mg/ml solution for injection 3ml pre-filled pen (Novo Nordisk Ltd) |
| 54462 | Insulin biphasic isophane human emp 25:75; 100 units/ml Injection |
| 64460 | Insulin glargine 300units/ml solution for injection 1.5ml pre-filled disposable devices |
| 7393 | Insulin glargine 100units/ml solution for injection 3ml cartridges |
| 28183 | Hypurin Porcine Isophane 100units/ml suspension for injection 10ml vials (Wockhardt UK Ltd) |
| 14925 | Insulin isophane human vial 100unit/ml Sterile suspension injection |
| 63562 | Insulin degludec 100units/ml / Liraglutide 3.6mg/ml solution for injection 3ml pre-filled disposable devices |
| 34097 | Human initard 50/50 100unit/ml Injection (Novo Nordisk Ltd) |
| 47856 | Neuphane 100unit/ml Injection (Wellcome Medical Division) |
| 14330 | Insulin detemir 100units/ml solution for injection 3ml pre-filled disposable devices |
| 9503 | Hypurin Bovine Protamine Zinc 100units/ml suspension for injection 10ml vials (Wockhardt UK Ltd) |
| 30686 | Insulin isophane porcine 100units/ml suspension for injection 3ml cartridges |
| 9341 | Insulin biphasic isophane human prb 30:70; 100 units/ml Injection |
| 31258 | Insulin lispro biphasic 25/75 100units/ml suspension for injection 3ml pre-filled disposable devices |
| 76776 | Suliqua 100units/ml / 33micrograms/ml solution for injection 3ml pre-filled SoloStar pen (Sanofi) |
| 10243 | Humalog Mix25 100units/ml suspension for injection 3ml cartridges (Eli Lilly and Company Ltd) |
| 5501 | Insuman basal 100iu/ml Injection (Aventis Pharma) |
| 55517 | Insulin isophane human 100units/ml suspension for injection 10ml vials |
| 50633 | Lantus 100units/ml solution for injection 3ml cartridges (Necessity Supplies Ltd) |
| 7771 | Human protaphane penfill 100 100unit/ml Penfill (Novo Nordisk Ltd) |
| 64723 | Abasaglar KwikPen 100units/ml solution for injection 3ml pre-filled pen (Eli Lilly and Company Ltd) |
| 33167 | Insulin biphasic isophane human crb 25:75; 100 units/ml Injection |
| 52748 | Insulatard Penfill 100units/ml suspension for injection 3ml cartridges (Waymade Healthcare Plc) |
| 64987 | Abasaglar 100units/ml solution for injection 3ml cartridges (Eli Lilly and Company Ltd) |
| 42395 | Humalog Mix25 100units/ml suspension for injection 10ml vials (Eli Lilly and Company Ltd) |
| 3439 | Penmix 10/90 Pen (Novo Nordisk Ltd) |
| 11055 | Insulin biphasic isophane human pyr 20:80; 100 units/ml Injection |
| 74391 | Humalog Mix50 KwikPen 100units/ml suspension for injection 3ml pre-filled pen (Sigma Pharmaceuticals Plc) |
| 41834 | Insulin zinc suspension lente 100iu/ml Injection (Celltech Pharma Europe Ltd) |
| 4784 | Lentard mc 100unit/ml Injection (Novo Nordisk Ltd) |
| 10915 | Humaject m1 100iu/ml M1 pen (Eli Lilly and Company Ltd) |
| 6057 | Lantus 100iu/ml Injection (Aventis Pharma) |
| 66316 | Lantus 100units/ml solution for injection 10ml vials (Mawdsley-Brooks & Company Ltd) |
| 74071 | Humalog Mix25 KwikPen 100units/ml suspension for injection 3ml pre-filled pen (Waymade Healthcare Plc) |
| 8895 | Initard 50/50 100unit/ml Injection (Novo Nordisk Ltd) |
| 13837 | Insulin biphasic isophane human prb 10:90; 100 units/ml Injection |
| 5255 | Mixtard 10 penfill 100 100iu/ml Penfill (Novo Nordisk Ltd) |
| 28185 | Insulin lispro biphasic 25/75 100units/ml suspension for injection 3ml cartridges |
| 14340 | Hypurin Bovine Isophane 100units/ml suspension for injection 10ml vials (Wockhardt UK Ltd) |
| 11080 | Insulin isophane human prb 100iu/ml Injection |
| 20422 | Insuman comb 15 100iu/ml Injection (Aventis Pharma) |
| 21395 | Insulin biphasic isophane human pyr 40:60; 100 units/ml Injection |
| 56495 | Lantus 100units/ml solution for injection 3ml pre-filled OptiSet pen (Waymade Healthcare Plc) |
| 21374 | Insulin biphasic isophane human prb 40:60; 100 units/ml Injection |
| 74845 | Humulin I 100units/ml suspension for injection 10ml vials (Waymade Healthcare Plc) |
| 1805 | Mixtard 30/70 100unit/ml Injection (Novo Nordisk Ltd) |
| 36146 | Insulin lispro biphasic 50/50 100units/ml suspension for injection 3ml cartridges |
| 14270 | Humalog Mix25 Pen 100units/ml suspension for injection 3ml pre-filled pen (Eli Lilly and Company Ltd) |
| 74388 | Humalog Mix50 100units/ml suspension for injection 3ml cartridges (Sigma Pharmaceuticals Plc) |
| 4715 | Humalog mix 25 25/75 100units/ml Injection (Eli Lilly and Company Ltd) |
| 7237 | Lantus 100units/ml solution for injection 3ml pre-filled OptiSet pen (Sanofi) |
| 79358 | Insulin biphasic isophane porcine 100 units/ml Injection |
| 12299 | Semitard mc 100unit/ml Injection (Novo Nordisk Ltd) |
| 76777 | Suliqua 100units/ml / 50micrograms/ml solution for injection 3ml pre-filled SoloStar pen (Sanofi) |
| 71361 | Humulin I 100units/ml suspension for injection 3ml cartridges (Waymade Healthcare Plc) |
| 49831 | Lantus 100units/ml solution for injection 3ml pre-filled SoloStar pen (Necessity Supplies Ltd) |
| 59500 | Insulin isophane human 100units/ml suspension for injection 5ml vials |
| 7772 | Human protaphane 100unit/ml Injection (Novo Nordisk Ltd) |
| 1595 | Insulatard NovoLet 100units/ml suspension for injection (Novo Nordisk Ltd) |
| 1886 | Insulatard 100iu/ml GE injection (Novo Nordisk Ltd) |
| 7402 | Lantus 100units/ml solution for injection 10ml vials (Sanofi) |
| 27177 | Insulin biphasic lispro human prb 50:50; 100 units/ml Injection |
| 76163 | Lantus 100units/ml solution for injection 3ml cartridges (Sigma Pharmaceuticals Plc) |
| 15961 | Insulin isophane human crb 100iu/ml Injection |
| 10184 | Insulin detemir 100 iu/ml Solution for injection |
| 71118 | Insulatard 100units/ml suspension for injection 10ml vials (Waymade Healthcare Plc) |
| 46001 | Insuman Basal 100units/ml suspension for injection 3ml pre-filled SoloStar pen (Sanofi) |
| 77284 | Insulin isophane human 100iu/ml Injection |
| 5891 | Insulatard FlexPen 100units/ml suspension for injection (Novo Nordisk Ltd) |
| 17712 | Hypurin Bovine Lente 100units/ml suspension for injection 10ml vials (Wockhardt UK Ltd) |
| 14357 | Humulin I 100units/ml suspension for injection 3ml cartridges (Eli Lilly and Company Ltd) |
| 13416 | Insulin biphasic 100 units/ml Injection |
| 14933 | Hypurin Porcine Isophane 100units/ml suspension for injection 3ml cartridges (Wockhardt UK Ltd) |
| 38422 | Isophane 100iu/ml Injection (Celltech Pharma Europe Ltd) |
| 27614 | Penmix 30/70 100iu/ml Injection (Novo Nordisk Ltd) |
| 21347 | Penmix 40/60 100iu/ml Injection (Novo Nordisk Ltd) |
| 25812 | Insulin isophane human 100units/ml suspension for injection 3ml pre-filled disposable devices |
| 3396 | Penmix 10/90 Penfill (Novo Nordisk Ltd) |
| 76728 | Semglee 100units/ml solution for injection 3ml pre-filled pen (Mylan) |
| 13516 | Hypurin bovine isophane 100unit/ml Injection (C P Pharmaceuticals Ltd) |
| 2220 | Penmix 20/80 Pen (Novo Nordisk Ltd) |
| 77794 | Toujeo 300units/ml solution for injection 3ml pre-filled DoubleStar pen (Sanofi) |
| 39006 | Humalog Mix25 KwikPen 100units/ml suspension for injection 3ml pre-filled pen (Eli Lilly and Company Ltd) |
| 10229 | Humulin I Pen 100units/ml suspension for injection 3ml pre-filled pen (Eli Lilly and Company Ltd) |
| 29837 | Insulin biphasic isophane human prb 25:75; 100 units/ml Injection |
| 76162 | Humulin I 100units/ml suspension for injection 3ml cartridges (Sigma Pharmaceuticals Plc) |
| 4790 | Mixtard 50 penfill 100 100iu/ml Penfill (Novo Nordisk Ltd) |
| 10208 | Insulatard InnoLet 100units/ml suspension for injection 3ml pre-filled pen (Novo Nordisk Ltd) |
| 12035 | Insulin zinc mixed bovine 100units/ml suspension for injection 10ml vials |
| 36031 | Insulin isophane biphasic porcine 30/70 100units/ml suspension for injection 3ml cartridges |
| 41120 | Insulin isophane biphasic human 50/50 100units/ml suspension for injection 3ml pre-filled disposable devices |
| 10277 | Humulin M3 100units/ml suspension for injection 3ml cartridges (Eli Lilly and Company Ltd) |
| 27280 | Insulin isophane biphasic porcine 30/70 100units/ml suspension for injection 10ml vials |
| 2221 | Mixtard 30 NovoLet 100units/ml suspension for injection (Novo Nordisk Ltd) |
| 76988 | NovoMix 30 Penfill 100units/ml suspension for injection 3ml cartridges (Sigma Pharmaceuticals Plc) |
| 67324 | Humulin M3 100units/ml suspension for injection 3ml cartridges (Waymade Healthcare Plc) |
| 31205 | Insuman Comb 50 100units/ml suspension for injection 3ml pre-filled OptiSet pen (Sanofi) |
| 76922 | Insulin isophane biphasic human 15/85 100units/ml suspension for injection 3ml pre-filled disposable devices |
| 24002 | Insuman Comb 25 100units/ml suspension for injection 5ml vials (Sanofi) |
| 60938 | Mixtard 30 100units/ml suspension for injection 10ml vials (Waymade Healthcare Plc) |
| 56489 | NovoMix 30 Penfill 100units/ml suspension for injection 3ml cartridges (Waymade Healthcare Plc) |
| 16160 | Humulin M3 Pen 100units/ml suspension for injection 3ml pre-filled pen (Eli Lilly and Company Ltd) |
| 5933 | Mixtard 50 NovoLet 100units/ml suspension for injection (Novo Nordisk Ltd) |
| 20995 | Hypurin Porcine 30/70 Mix 100units/ml suspension for injection 3ml cartridges (Wockhardt UK Ltd) |
| 74680 | NovoMix 30 Penfill 100units/ml suspension for injection 3ml cartridges (DE Pharmaceuticals) |
| 2459 | Pork Mixtard 30 100units/ml suspension for injection 10ml vials (Novo Nordisk Ltd) |
| 71369 | NovoMix 30 FlexPen 100units/ml suspension for injection 3ml pre-filled pen (Sigma Pharmaceuticals Plc) |
| 56857 | Insulin isophane biphasic human 15/85 100units/ml suspension for injection 3ml cartridges |
| 24800 | Hypurin Porcine 30/70 Mix 100units/ml suspension for injection 10ml vials (Wockhardt UK Ltd) |
| 36194 | Insulin isophane biphasic human 25/75 100units/ml suspension for injection 3ml cartridges |
| 19878 | Insulin isophane biphasic human 30/70 100units/ml suspension for injection 3ml pre-filled disposable devices |
| 44378 | Insulin isophane biphasic human 25/75 100units/ml suspension for injection 3ml pre-filled disposable devices |
| 16152 | Insulin isophane biphasic human 30/70 100units/ml suspension for injection 3ml cartridges |
| 35253 | Insuman Comb 50 100units/ml suspension for injection 3ml cartridges (Sanofi) |
| 67267 | Mixtard 30 InnoLet 100units/ml suspension for injection 3ml pre-filled pen (Waymade Healthcare Plc) |
| 7267 | NovoMix 30 Penfill 100units/ml suspension for injection 3ml cartridges (Novo Nordisk Ltd) |
| 43991 | Humulin M3 KwikPen 100units/ml suspension for injection 3ml pre-filled pen (Eli Lilly and Company Ltd) |
| 21232 | Insulin isophane biphasic human 30/70 100units/ml suspension for injection 10ml vials |
| 13277 | Mixtard 50 Penfill 100units/ml suspension for injection 3ml cartridges (Novo Nordisk Ltd) |
| 23099 | Insulin aspart biphasic 30/70 100units/ml suspension for injection 3ml pre-filled disposable devices |
| 24993 | Insuman Comb 25 100units/ml suspension for injection 3ml cartridges (Sanofi) |
| 28096 | Insulin isophane biphasic human 50/50 100units/ml suspension for injection 3ml cartridges |
| 60933 | Humulin M3 100units/ml suspension for injection 10ml vials (Sigma Pharmaceuticals Plc) |
| 44480 | Insuman Comb 25 100units/ml suspension for injection 3ml pre-filled SoloStar pen (Sanofi) |
| 5845 | Mixtard 30 InnoLet 100units/ml suspension for injection 3ml pre-filled pen (Novo Nordisk Ltd) |
| 7231 | Mixtard 30 Penfill 100units/ml suspension for injection 3ml cartridges (Novo Nordisk Ltd) |
| 7228 | NovoMix 30 FlexPen 100units/ml suspension for injection 3ml pre-filled pen (Novo Nordisk Ltd) |
| 30819 | Insuman Comb 15 100units/ml suspension for injection 3ml pre-filled OptiSet pen (Sanofi) |
| 67266 | Mixtard 50 Penfill 100units/ml suspension for injection 3ml cartridges (Waymade Healthcare Plc) |
| 45158 | Insuman Comb 15 100units/ml suspension for injection 3ml cartridges (Sanofi) |
| 7300 | Mixtard 30 100units/ml suspension for injection 10ml vials (Novo Nordisk Ltd) |
| 7793 | HumaJect M3 Pen 100units/ml suspension for injection (Eli Lilly and Company Ltd) |
| 74842 | Humulin M3 100units/ml suspension for injection 3ml cartridges (Mawdsley-Brooks & Company Ltd) |
| 24795 | Insulin aspart biphasic 30/70 100units/ml suspension for injection 3ml cartridges |
| 74020 | NovoMix 30 Penfill 100units/ml suspension for injection 3ml cartridges (Mawdsley-Brooks & Company Ltd) |
| 25133 | Insuman Comb 25 100units/ml suspension for injection 3ml pre-filled OptiSet pen (Sanofi) |
| 71340 | Mixtard 30 Penfill 100units/ml suspension for injection 3ml cartridges (Waymade Healthcare Plc) |
| 42954 | Insulin isophane biphasic human 25/75 100units/ml suspension for injection 5ml vials |
| 19513 | Humulin M3 100units/ml suspension for injection 10ml vials (Eli Lilly and Company Ltd) |
| 57620 | Humulin M3 100units/ml suspension for injection 10ml vials (Mawdsley-Brooks & Company Ltd) |
| 53710 | Insulin human 500units/ml solution for injection 20ml vials |
| 60951 | Insulin human 100units/ml solution for injection 10ml vials |
| 62276 | Humulin R 500units/ml solution for injection 20ml vials (Imported (United States)) |
| 79254 | Insulin human 500units/ml solution for injection 3ml pre-filled disposable devices |
| 72479 | Humulin R KwikPen 500units/ml solution for injection 3ml pre-filled pen (Imported (United States)) |
| 67429 | Insulin human 100units/ml solution for injection 3.15ml cartridges |
| 77498 | Gliclazide 160mg tablets |
| 5276 | Glimepiride 1mg tablets |
| 44250 | Metformin 500mg/5ml Oral solution (Hillcross Pharmaceuticals Ltd) |
| 2219 | Glibenclamide 2.5mg tablets |
| 68415 | Gliclazide 30mg modified-release tablets (Phoenix Healthcare Distribution Ltd) |
| 62034 | Laaglyda MR 60mg tablets (Consilient Health Ltd) |
| 7912 | Semi-Daonil 2.5mg tablets (Sanofi) |
| 43684 | Janumet 50mg/1000mg tablets (Merck Sharp & Dohme Ltd) |
| 63823 | Dulaglutide 1.5mg/0.5ml solution for injection pre-filled disposable devices |
| 77437 | Amaryl 1mg tablets (Sigma Pharmaceuticals Plc) |
| 3740 | Guar gum 90% granules |
| 39598 | Metformin 1g modified-release tablets |
| 54480 | Forxiga 5mg tablets (AstraZeneca UK Ltd) |
| 69540 | Saxagliptin 5mg / Dapagliflozin 10mg tablets |
| 39560 | Bolamyn SR 500mg tablets (Teva UK Ltd) |
| 30316 | Metformin with pioglitazone 850mg + 15mg Tablet |
| 53288 | Gliclazide 30mg modified-release tablets (A A H Pharmaceuticals Ltd) |
| 34957 | Tolbutamide 500mg tablets (A A H Pharmaceuticals Ltd) |
| 57830 | Gliclazide 30mg modified-release tablets (Alliance Healthcare (Distribution) Ltd) |
| 57147 | Bolamyn SR 1000mg tablets (Teva UK Ltd) |
| 13628 | Romozin 400mg Tablet (Glaxo Wellcome UK Ltd) |
| 50087 | Januvia 50mg tablets (Merck Sharp & Dohme Ltd) |
| 12513 | Glibenese 5mg tablets (Pfizer Ltd) |
| 68589 | Metformin 1g/5ml oral solution sugar free |
| 11604 | Rosiglitazone 1mg / Metformin 500mg tablets |
| 16602 | Calabren 2.5mg Tablet (Berk Pharmaceuticals Ltd) |
| 46989 | Metabet SR 1000mg tablets (Morningside Healthcare Ltd) |
| 59385 | Vipdomet 12.5mg/1000mg tablets (Takeda UK Ltd) |
| 26118 | Dimelor 500mg Tablet (Eli Lilly and Company Ltd) |
| 70463 | Synjardy 12.5mg/850mg tablets (Boehringer Ingelheim Ltd) |
| 21892 | Diaglyk 80mg tablets (Ashbourne Pharmaceuticals Ltd) |
| 44473 | Edicil MR 30mg tablets (Teva UK Ltd) |
| 1847 | Chlorpropamide 250mg tablets |
| 51135 | Metformin 500mg modified-release tablets (A A H Pharmaceuticals Ltd) |
| 1965 | Tolbutamide 500mg tablets |
| 62265 | Metformin 500mg modified-release tablets (Mawdsley-Brooks & Company Ltd) |
| 60074 | Metformin 1g modified-release tablets (Waymade Healthcare Plc) |
| 72539 | Sitagliptin 50mg/5ml oral solution |
| 46469 | Bydureon 2mg powder and solvent for prolonged-release suspension for injection vials (AstraZeneca UK Ltd) |
| 32 | Gliclazide 80mg tablets |
| 71020 | Acarbose 50mg tablets (Actavis UK Ltd) |
| 69548 | Saxenda 6mg/ml solution for injection 3ml pre-filled pen (Novo Nordisk Ltd) |
| 26218 | Calabren 5mg Tablet (Berk Pharmaceuticals Ltd) |
| 15374 | Gliclazide 40mg/5ml oral suspension |
| 50570 | Glucophage SR 500mg tablets (Lexon (UK) Ltd) |
| 34917 | Metformin 500mg tablets (Teva UK Ltd) |
| 42161 | Orabet 500mg Tablet (Sandoz Ltd) |
| 11695 | Diamicron 30mg MR tablets (Servier Laboratories Ltd) |
| 41558 | Glibenclamide 5mg tablets (Teva UK Ltd) |
| 63031 | Dapagliflozin 5mg / Metformin 850mg tablets |
| 21424 | Glibenclamide 5mg/5ml oral suspension |
| 45215 | Gliclazide 80mg Tablet (Neo Laboratories Ltd) |
| 4862 | Diabetamide 2.5mg tablets (Ashbourne Pharmaceuticals Ltd) |
| 63046 | Pioglitazone 45mg tablets (A A H Pharmaceuticals Ltd) |
| 31212 | Gliclazide 80mg tablets (Actavis UK Ltd) |
| 48149 | Metformin 500mg tablets (Almus Pharmaceuticals Ltd) |
| 11284 | Amaryl 4mg tablets (Zentiva) |
| 74860 | NovoNorm 1mg tablets (Waymade Healthcare Plc) |
| 56831 | Troglitazone 200mg Tablet |
| 58865 | Komboglyze 2.5mg/850mg tablets (AstraZeneca UK Ltd) |
| 62661 | Bydureon 2mg powder and solvent for prolonged-release suspension for injection pre-filled pen (AstraZeneca UK Ltd) |
| 11609 | Metformin with rosiglitazone 500mg + 1mg Tablet |
| 11737 | Metformin with rosiglitazone 1000mg + 4mg Tablet |
| 54203 | Forxiga 10mg tablets (AstraZeneca UK Ltd) |
| 34004 | Metformin 500mg tablets (IVAX Pharmaceuticals UK Ltd) |
| 43065 | Gliclazide 40mg tablets |
| 34507 | Glibenclamide 2.5mg tablets (Wockhardt UK Ltd) |
| 49738 | Metformin 1g modified-release tablets (A A H Pharmaceuticals Ltd) |
| 20889 | Actos 30mg tablets (Takeda UK Ltd) |
| 55739 | Metformin 500mg tablets (Tillomed Laboratories Ltd) |
| 72107 | Meijumet 500mg modified-release tablets (Medreich Plc) |
| 55459 | Lixisenatide 10micrograms/0.2ml solution for injection 3ml pre-filled disposable devices |
| 735 | Metformin 100mg/ml Oral solution |
| 548 | Pioglitazone 15mg tablets |
| 38551 | Eucreas 50mg/1000mg tablets (Novartis Pharmaceuticals UK Ltd) |
| 60682 | Vipidia 25mg tablets (Takeda UK Ltd) |
| 37874 | Vildagliptin 50mg / Metformin 850mg tablets |
| 75397 | Semaglutide 0.25mg/0.19ml solution for injection 1.5ml pre-filled disposable device |
| 75740 | Ozempic 0.25mg/0.19ml solution for injection 1.5ml pre-filled pen (Novo Nordisk Ltd) |
| 68819 | Gliclazide 80mg tablets (Bristol Laboratories Ltd) |
| 35144 | Byetta 5micrograms/0.02ml solution for injection 1.2ml pre-filled pen (AstraZeneca UK Ltd) |
| 68389 | Metformin 500mg/5ml oral solution sugar free (Pinewood Healthcare) |
| 40693 | Liraglutide 6mg/ml solution for injection 3ml pre-filled disposable devices |
| 61311 | Glimepiride 4mg tablets (Sigma Pharmaceuticals Plc) |
| 7166 | Glucophage 500mg tablets (Merck Serono Ltd) |
| 8034 | Diabinese 100mg Tablet (Pfizer Ltd) |
| 40110 | Glucophage 500mg oral powder sachets (Merck Serono Ltd) |
| 68214 | Metformin 500mg/5ml oral solution sugar free (A A H Pharmaceuticals Ltd) |
| 9865 | Repaglinide 500microgram tablets |
| 63516 | Forxiga 10mg tablets (Waymade Healthcare Plc) |
| 34697 | Metformin 850mg tablets (Wockhardt UK Ltd) |
| 54891 | Saxagliptin 2.5mg / Metformin 1g tablets |
| 9707 | Repaglinide 1mg tablets |
| 12259 | Glibornuride 25mg Tablet |
| 61756 | Empagliflozin 10mg tablets |
| 17343 | Gliclazide 80mg tablets (A A H Pharmaceuticals Ltd) |
| 11601 | Rosiglitazone 2mg / Metformin 500mg tablets |
| 69370 | Synjardy 5mg/850mg tablets (Boehringer Ingelheim Ltd) |
| 57457 | Metformin 500mg tablets (Milpharm Ltd) |
| 52449 | Linagliptin 2.5mg / Metformin 850mg tablets |
| 4307 | Guarina Sachets (Norgine Pharmaceuticals Ltd) |
| 68934 | Diabiom 30mg tablets (Tillomed Laboratories Ltd) |
| 25678 | Glucamet 500mg Tablet (Opus Pharmaceuticals Ltd) |
| 46927 | Tolbutamide 500mg tablets (Teva UK Ltd) |
| 39149 | Galvus 50mg tablets (Novartis Pharmaceuticals UK Ltd) |
| 34802 | Glipizide 5mg tablets (IVAX Pharmaceuticals UK Ltd) |
| 56437 | Gliclazide 60mg modified-release tablets |
| 41431 | Onglyza 5mg tablets (AstraZeneca UK Ltd) |
| 69669 | Gliclazide 160mg/5ml oral suspension |
| 71781 | Gliclazide 40mg tablets (Teva UK Ltd) |
| 43465 | Zicron 40mg tablets (Bristol Laboratories Ltd) |
| 41593 | Glibenclamide 2.5mg tablets (Teva UK Ltd) |
| 68258 | Alogliptin 25mg tablets (Colorama Pharmaceuticals Ltd) |
| 7409 | Amaryl 3mg tablets (Zentiva) |
| 9699 | Pioglitazone 30mg tablets |
| 58607 | Metformin 500mg/5ml oral solution sugar free (Zentiva) |
| 40233 | Metformin 1g oral powder sachets sugar free |
| 34135 | Metformin 500mg Tablet (M & A Pharmachem Ltd) |
| 52634 | Glucophage SR 500mg tablets (DE Pharmaceuticals) |
| 479 | Acarbose 50mg tablets |
| 78125 | Gliclazide 30mg modified-release tablets (Sigma Pharmaceuticals Plc) |
| 38355 | Metformin 750mg modified-release tablets |
| 62326 | Vipidia 6.25mg tablets (Takeda UK Ltd) |
| 77978 | Januvia 50mg tablets (Pharmaram Ltd) |
| 59620 | Glucophage SR 500mg tablets (Waymade Healthcare Plc) |
| 56965 | Komboglyze 2.5mg/1000mg tablets (AstraZeneca UK Ltd) |
| 35150 | Byetta 10micrograms/0.04ml solution for injection 2.4ml pre-filled pen (AstraZeneca UK Ltd) |
| 76624 | Semaglutide 1mg/0.74ml solution for injection 3ml pre-filled disposable device |
| 15955 | Starlix 120mg tablets (Novartis Pharmaceuticals UK Ltd) |
| 60430 | Invokana 100mg tablets (Napp Pharmaceuticals Ltd) |
| 27125 | Starlix 180mg tablets (Novartis Pharmaceuticals UK Ltd) |
| 48533 | Sitagliptin 25mg tablets |
| 5627 | Gliclazide 30mg modified-release tablets |
| 73525 | Metformin 500mg/5ml oral solution sugar free (Alliance Healthcare (Distribution) Ltd) |
| 34399 | Gliclazide 80mg tablets (IVAX Pharmaceuticals UK Ltd) |
| 69947 | Albiglutide 30mg powder and solvent for solution for injection pre-filled disposable devices |
| 80087 | Empagliflozin 25mg / Linagliptin 5mg tablets |
| 6337 | Glimepiride 3mg tablets |
| 53774 | Metabet SR 500mg tablets (Actavis UK Ltd) |
| 34598 | Metformin 500mg tablets (Mylan) |
| 63045 | Metformin 850mg tablets (Relonchem Ltd) |
| 58882 | Gliclazide 120mg/5ml oral suspension |
| 40642 | Victoza 6mg/ml solution for injection 3ml pre-filled pen (Novo Nordisk Ltd) |
| 23 | Metformin 500mg tablets |
| 70744 | Metformin 500mg/5ml oral solution sugar free (Advanz Pharma) |
| 7375 | Rosiglitazone 4mg / Metformin 1g tablets |
| 21564 | Gliclazide 80mg tablets (Wockhardt UK Ltd) |
| 71477 | Glimepiride 1mg tablets (Alliance Healthcare (Distribution) Ltd) |
| 1254 | Glibenclamide 5mg tablets |
| 16044 | Glucophage SR 500mg tablets (Merck Serono Ltd) |
| 60012 | Dapagliflozin 5mg / Metformin 1g tablets |
| 71890 | Metformin 500mg/5ml oral solution sugar free (Almus Pharmaceuticals Ltd) |
| 57601 | Daonil 5mg tablets (Dowelhurst Ltd) |
| 469 | Rosiglitazone 4mg tablets |
| 5678 | Nateglinide 120mg tablets |
| 71665 | Glimepiride 3mg tablets (Alliance Healthcare (Distribution) Ltd) |
| 76021 | Ozempic 1mg/0.74ml solution for injection 3ml pre-filled pen (Novo Nordisk Ltd) |
| 78946 | Ertugliflozin 15mg tablets |
| 54973 | Saxagliptin 2.5mg / Metformin 850mg tablets |
| 54150 | Jentadueto 2.5mg/850mg tablets (Boehringer Ingelheim Ltd) |
| 62904 | Exenatide 2mg powder and solvent for prolonged-release suspension for injection pre-filled disposable devices |
| 72163 | Pioglitazone 45mg tablets (Alliance Healthcare (Distribution) Ltd) |
| 24848 | Glymidine sodium 500mg Tablet |
| 69654 | Qtern 5mg/10mg tablets (AstraZeneca UK Ltd) |
| 11990 | Metformin 500mg/5ml oral solution sugar free |
| 65563 | Pioglitazone 15mg tablets (Alliance Healthcare (Distribution) Ltd) |
| 38400 | Glucophage SR 750mg tablets (Merck Serono Ltd) |
| 34323 | Metformin 500mg tablets (A A H Pharmaceuticals Ltd) |
| 74021 | Metformin 250mg/5ml oral solution |
| 17698 | Minodiab 5mg tablets (Pfizer Ltd) |
| 48120 | Avandia 2mg Tablet (GlaxoSmithKline UK Ltd) |
| 5227 | Rosiglitazone 8mg tablets |
| 74734 | Metformin 850mg capsules |
| 73303 | Metformin 500mg/5ml oral solution sugar free (Colonis Pharma Ltd) |
| 65083 | Synjardy 5mg/1000mg tablets (Boehringer Ingelheim Ltd) |
| 7284 | Amaryl 2mg tablets (Zentiva) |
| 69221 | Metformin 1g oral powder sachets sugar free (J M McGill Ltd) |
| 34676 | Glibenclamide 2.5mg tablets (A A H Pharmaceuticals Ltd) |
| 71012 | Metformin 500mg tablets (Phoenix Healthcare Distribution Ltd) |
| 21489 | Tolanase 250mg Tablet (Pharmacia Ltd) |
| 34706 | Glibenclamide 2.5mg tablets (IVAX Pharmaceuticals UK Ltd) |
| 27969 | Glymese 250mg Tablet (DDSA Pharmaceuticals Ltd) |
| 11760 | Metformin with rosiglitazone 1000mg + 2mg Tablet |
| 70432 | Bilxona 30mg modified-release tablets (Accord Healthcare Ltd) |
| 45821 | Onglyza 2.5mg tablets (AstraZeneca UK Ltd) |
| 55413 | Lixisenatide 20micrograms/0.2ml solution for injection 3ml pre-filled disposable devices |
| 66399 | Glimepiride 2mg tablets (A A H Pharmaceuticals Ltd) |
| 7610 | Glucophage 850mg tablets (Merck Serono Ltd) |
| 80647 | Metuxtan SR 500mg tablets (Accord Healthcare Ltd) |
| 55862 | Gliclazide Oral solution |
| 60495 | Gliclazide 80mg tablets (Teva UK Ltd) |
| 40007 | Glucophage 1000mg oral powder sachets (Merck Serono Ltd) |
| 77753 | Meijumet 1000mg modified-release tablets (Medreich Plc) |
| 53867 | Metformin 500mg tablets (Zentiva) |
| 78258 | Pioglitazone 15mg/5ml oral suspension |
| 41559 | Glibenclamide 5mg tablets (A A H Pharmaceuticals Ltd) |
| 61957 | Gliclazide 40mg tablets (A A H Pharmaceuticals Ltd) |
| 62605 | Metformin 850mg tablets (Kent Pharmaceuticals Ltd) |
| 52442 | Metformin 500mg tablets (Pfizer Ltd) |
| 5636 | Glipizide 5mg tablets |
| 77762 | Ertugliflozin 5mg tablets |
| 78392 | Metformin 850mg tablets (Zentiva) |
| 55728 | Lyxumia 10micrograms/0.2ml solution for injection 3ml pre-filled pen (Sanofi) |
| 63421 | Pioglitazone 30mg tablets (Teva UK Ltd) |
| 61925 | NovoNorm 500microgram tablets (Waymade Healthcare Plc) |
| 34836 | Metformin 850mg tablets (Actavis UK Ltd) |
| 77291 | Gliclazide 80mg tablets (Kent Pharmaceuticals Ltd) |
| 63107 | Pioglitazone 45mg tablets (Waymade Healthcare Plc) |
| 62426 | Pioglitazone 30mg tablets (Accord Healthcare Ltd) |
| 57659 | Pioglitazone 30mg tablets (Actavis UK Ltd) |
| 11321 | NovoNorm 1mg tablets (Novo Nordisk Ltd) |
| 68289 | Glimepiride 4mg tablets (Waymade Healthcare Plc) |
| 78293 | Alogliptin 12.5mg / Metformin 1g tablets (Colorama Pharmaceuticals Ltd) |
| 10051 | Pioglitazone 45mg tablets |
| 70490 | Bilxona 60mg modified-release tablets (Actavis UK Ltd) |
| 65066 | Empagliflozin 12.5mg / Metformin 1g tablets |
| 51080 | Metabet SR 1000mg tablets (Actavis UK Ltd) |
| 50970 | Metformin 500mg tablets (Bristol Laboratories Ltd) |
| 19336 | Tolazamide 100mg Tablet |
| 56376 | Rosiglitazone 4mg with glimepiride 4mg tablet |
| 25636 | Libanil 2.5mg Tablet (Approved Prescription Services Ltd) |
| 8976 | Euglucon 2.5mg tablets (Aventis Pharma) |
| 31146 | Metsol 500mg/5ml oral solution (Kappin Ltd) |
| 75823 | Gliclazide 40mg tablets (Waymade Healthcare Plc) |
| 35462 | Januvia 100mg tablets (Merck Sharp & Dohme Ltd) |
| 27501 | Orabet 500mg Tablet (Lagap) |
| 37902 | Vildagliptin 50mg / Metformin 1g tablets |
| 31474 | Libanil 5mg Tablet (Approved Prescription Services Ltd) |
| 7695 | Guarem Sachets (Shire Pharmaceuticals Ltd) |
| 79017 | Metformin 850mg tablets (Mylan) |
| 5989 | Nateglinide 180mg tablets |
| 40425 | Nazdol MR 30mg tablets (Teva UK Ltd) |
| 11483 | Nateglinide 60mg tablets |
| 50124 | Januvia 25mg tablets (Merck Sharp & Dohme Ltd) |
| 72852 | Gliclazide 40mg tablets (Accord Healthcare Ltd) |
| 41204 | Saxagliptin 5mg tablets |
| 36774 | Prandin 1mg tablets (Novo Nordisk Ltd) |
| 54442 | Metformin (roi) 1000mg Tablet |
| 60643 | Xigduo 5mg/1000mg tablets (AstraZeneca UK Ltd) |
| 55729 | Lyxumia 20micrograms/0.2ml solution for injection 3ml pre-filled pen (Sanofi) |
| 73254 | Yaltormin SR 1000mg tablets (Wockhardt UK Ltd) |
| 72695 | Metformin 500mg Tablet (Celltech Pharma Europe Ltd) |
| 79372 | Glibenclamide 5mg Tablet (Celltech Pharma Europe Ltd) |
| 65344 | Empagliflozin 5mg / Metformin 850mg tablets |
| 34742 | Metformin 850mg tablets (Teva UK Ltd) |
| 50821 | Metformin 850mg tablets (Pfizer Ltd) |
| 10427 | Tolazamide 250mg Tablet |
| 70477 | Glucient SR 750mg tablets (Consilient Health Ltd) |
| 71476 | Glimepiride 2mg tablets (Alliance Healthcare (Distribution) Ltd) |
| 34932 | Gliclazide 80mg tablets (Genus Pharmaceuticals Ltd) |
| 48139 | Pioglitazone 30mg tablets (A A H Pharmaceuticals Ltd) |
| 68675 | Glimepiride 4mg tablets (Somex Pharma) |
| 11366 | NovoNorm 2mg tablets (Novo Nordisk Ltd) |
| 34504 | Metformin 500mg tablets (Wockhardt UK Ltd) |
| 34020 | Metformin 850mg tablets (IVAX Pharmaceuticals UK Ltd) |
| 54265 | Dapagliflozin 5mg tablets |
| 60497 | Alogliptin 12.5mg / Metformin 1g tablets |
| 1964 | Diamicron 80mg tablets (Servier Laboratories Ltd) |
| 48401 | Sitagliptin 50mg tablets |
| 63336 | Trulicity 1.5mg/0.5ml solution for injection pre-filled pen (Eli Lilly and Company Ltd) |
| 54182 | Dapagliflozin 10mg tablets |
| 61559 | Sukkarto SR 500mg tablets (Morningside Healthcare Ltd) |
| 35251 | Exenatide 5micrograms/0.02ml solution for injection 1.2ml pre-filled disposable devices |
| 7332 | Amaryl 1mg tablets (Zentiva) |
| 9105 | Glucobay 100mg tablets (Bayer Plc) |
| 63048 | Gliclazide 80mg tablets (Alliance Healthcare (Distribution) Ltd) |
| 46665 | Linagliptin 5mg tablets |
| 34563 | Glibenclamide 5mg tablets (Wockhardt UK Ltd) |
| 75938 | Ozempic 0.5mg/0.37ml solution for injection 1.5ml pre-filled pen (Novo Nordisk Ltd) |
| 35149 | Exenatide 10micrograms/0.04ml solution for injection 2.4ml pre-filled disposable devices |
| 23945 | Starlix 60mg tablets (Novartis Pharmaceuticals UK Ltd) |
| 55767 | Lyxumia 10micrograms/20micrograms treatment initiation pack (Sanofi) |
| 62760 | Jardiance 10mg tablets (Boehringer Ingelheim Ltd) |
| 29939 | Gliclazide 80mg tablets (Mylan) |
| 48056 | Gliclazide 80mg tablets (Sovereign Medical Ltd) |
| 36856 | Gliclazide 80mg tablets (Sandoz Ltd) |
| 63307 | Metformin 1g/5ml oral solution |
| 54898 | Metformin 850mg tablets (Almus Pharmaceuticals Ltd) |
| 78537 | Metformin 850mg/5ml oral suspension |
| 46458 | Exenatide 2mg powder and solvent for prolonged-release suspension for injection vials |
| 18220 | Pioglitazone 15mg / Metformin 850mg tablets |
| 35022 | Sitagliptin 100mg tablets |
| 51955 | Gliclazide 80mg tablets (Accord Healthcare Ltd) |
| 65562 | Pioglitazone 30mg tablets (Alliance Healthcare (Distribution) Ltd) |
| 30460 | Malix 5mg Tablet (Lagap) |
| 60968 | Metformin 500mg modified-release tablets (Actavis UK Ltd) |
| 29326 | Glipizide 5mg tablets (Mylan) |
| 47939 | Glucient SR 500mg tablets (Consilient Health Ltd) |
| 65057 | Empagliflozin 5mg / Metformin 1g tablets |
| 55723 | Lixisenatide 10micrograms/0.2ml solution for injection 3ml pre-filled disposable devices and Lixisenatide 20micrograms/0.2ml solution for injection 3ml pre-filled disposable devices |
| 73252 | Yaltormin SR 500mg tablets (Wockhardt UK Ltd) |
| 64900 | Glidipion 30mg tablets (Actavis UK Ltd) |
| 60379 | Invokana 300mg tablets (Napp Pharmaceuticals Ltd) |
| 37875 | Vildagliptin 50mg tablets |
| 62824 | Metformin 1g modified-release tablets (Actavis UK Ltd) |
| 49502 | Glucophage SR 500mg tablets (Mawdsley-Brooks & Company Ltd) |
| 45581 | Metabet SR 500mg tablets (Morningside Healthcare Ltd) |
| 11316 | NovoNorm 500microgram tablets (Novo Nordisk Ltd) |
| 50682 | Jentadueto 2.5mg/1000mg tablets (Boehringer Ingelheim Ltd) |
| 42790 | Gliclazide 80mg Tablet (Merck Generics (UK) Ltd) |
| 67781 | Gliclazide 80mg tablets (Milpharm Ltd) |
| 60386 | Canagliflozin 300mg tablets |
| 44738 | Niddaryl 1mg tablets (Dee Pharmaceuticals Ltd) |
| 64217 | Jardiance 25mg tablets (Boehringer Ingelheim Ltd) |
| 71198 | Metformin 500mg tablets (Zanza Specials International Ltd) |
| 73808 | Metformin 500mg/5ml oral solution sugar free (Actavis UK Ltd) |
| 65694 | Metformin 500mg modified-release tablets (Waymade Healthcare Plc) |
| 33674 | Metformin 850mg tablets (A A H Pharmaceuticals Ltd) |
| 31077 | Competact 15mg/850mg tablets (Takeda UK Ltd) |
| 20287 | Actos 15mg tablets (Takeda UK Ltd) |
| 13331 | Euglucon 5mg tablets (Sanofi) |
| 63401 | Trulicity 0.75mg/0.5ml solution for injection pre-filled pen (Eli Lilly and Company Ltd) |
| 5174 | Acarbose 100mg tablets |
| 33087 | Metformin 500mg tablets (Actavis UK Ltd) |
| 5316 | Glimepiride 4mg tablets |
| 64939 | Glucient SR 1000mg tablets (Consilient Health Ltd) |
| 12245 | Glutril 25mg Tablet (Roche Products Ltd) |
| 65059 | Xigduo 5mg/850mg tablets (AstraZeneca UK Ltd) |
| 75700 | Metformin 500mg/5ml oral solution sugar free (Waymade Healthcare Plc) |
| 22145 | Tolanase 100mg Tablet (Pharmacia Ltd) |
| 7744 | Daonil 5mg tablets (Sanofi) |
| 60681 | Vipidia 12.5mg tablets (Takeda UK Ltd) |
| 39203 | Eucreas 50mg/850mg tablets (Novartis Pharmaceuticals UK Ltd) |
| 71667 | Glimepiride 1mg tablets (Teva UK Ltd) |
| 73511 | Yaltormin SR 750mg tablets (Wockhardt UK Ltd) |
| 63785 | Dulaglutide 0.75mg/0.5ml solution for injection pre-filled disposable devices |
| 72046 | Metformin 500mg tablets (DE Pharmaceuticals) |
| 60286 | Metformin 500mg/5ml oral suspension |
| 56208 | Pioglitazone 15mg tablets (A A H Pharmaceuticals Ltd) |
| 11610 | Metformin with rosiglitazone 500mg + 2mg Tablet |
| 43270 | Metformin 500mg/5ml oral solution sugar free (Rosemont Pharmaceuticals Ltd) |
| 54764 | Gliclazide 80mg tablets (Arrow Generics Ltd) |
| 78538 | Guar gum mini tablets |
| 5353 | Glimepiride 2mg tablets |
| 66854 | Vokanamet 50mg/1000mg tablets (Napp Pharmaceuticals Ltd) |
| 65923 | Metformin 1g modified-release tablets (Mawdsley-Brooks & Company Ltd) |
| 55711 | Metformin 500mg tablets (Alliance Healthcare (Distribution) Ltd) |
| 56008 | Gliclazide 80mg tablets (Almus Pharmaceuticals Ltd) |
| 66136 | Glucophage SR 1000mg tablets (Waymade Healthcare Plc) |
| 5621 | Glucobay 50mg tablets (Bayer Plc) |
| 9748 | Repaglinide 2mg tablets |
| 72001 | Metformin 1g modified-release tablets (DE Pharmaceuticals) |
| 1253 | Chlorpropamide 100mg tablets |
| 93 | Metformin 850mg tablets |
| 11717 | Rosiglitazone 2mg / Metformin 1g tablets |
| 79854 | Pioglitazone 15mg tablets (Accord Healthcare Ltd) |
| 60211 | Canagliflozin 100mg tablets |
| 75778 | Metformin 500mg tablets (Crescent Pharma Ltd) |
| 46716 | Trajenta 5mg tablets (Boehringer Ingelheim Ltd) |
| 66855 | Empagliflozin 12.5mg / Metformin 850mg tablets |
| 51527 | Metformin 500mg tablets (Boston Healthcare Ltd) |
| 61043 | Sukkarto SR 1000mg tablets (Morningside Healthcare Ltd) |
| 67056 | Amaryl 1mg tablets (Lexon (UK) Ltd) |
| 62144 | Metformin 500mg modified-release tablets (DE Pharmaceuticals) |
| 43619 | Metformin 1g / Sitagliptin 50mg tablets |
| 8168 | Diabinese 250mg Tablet (Pfizer Ltd) |
| 55270 | Duformin 500mg Tablet (Dumex Ltd) |
| 53478 | Metformin 500mg modified-release tablets (Kent Pharmaceuticals Ltd) |
| 52445 | Linagliptin 2.5mg / Metformin 1g tablets |
| 80319 | Empagliflozin 10mg / Linagliptin 5mg tablets |
| 59177 | Alogliptin 25mg tablets |
| 68203 | Metformin 500mg modified-release tablets (Almus Pharmaceuticals Ltd) |
| 58051 | Metformin 500mg/5ml oral solution |
| 52203 | Enyglid 0.5mg tablets (Consilient Health Ltd) |
| 12455 | Rastinon 500mg Tablet (Hoechst Marion Roussel) |
| 33673 | Tolbutamide 500mg tablets (Actavis UK Ltd) |
| 63131 | Ziclaseg 30mg modified-release tablets (Lupin Healthcare (UK) Ltd) |
| 62172 | Empagliflozin 25mg tablets |
| 28708 | Malix 2.5mg Tablet (Lagap) |
| 75008 | Glibenclamide 2.5mg Tablet (Berk Pharmaceuticals Ltd) |
| 35561 | Prandin 2mg tablets (Novo Nordisk Ltd) |
| 47074 | Gliclazide 80mg/5ml oral suspension |
| 39729 | Glucophage SR 1000mg tablets (Merck Serono Ltd) |
| 22858 | Acetohexamide 500mg tablets |
| 69885 | Pioglitazone 30mg tablets (Consilient Health Ltd) |
| 45775 | Saxagliptin 2.5mg tablets |
| 74051 | Onglyza 5mg tablets (Sigma Pharmaceuticals Plc) |
| 64622 | Bydureon 2mg powder and solvent for prolonged-release suspension for injection vials (Lexon (UK) Ltd) |
| 52221 | Diagemet XL 500mg tablets (Thornton & Ross Ltd) |
| 36948 | Prandin 0.5mg tablets (Novo Nordisk Ltd) |
| 73150 | Sitagliptin 100mg tablets (Waymade Healthcare Plc) |
| 69459 | Alogliptin 25mg tablets (Ennogen Healthcare Ltd) |
| 62014 | Glimepiride 2mg tablets (Accord Healthcare Ltd) |
| 64743 | Canagliflozin 50mg / Metformin 850mg tablets |
| 26258 | Glucamet 850mg Tablet (Opus Pharmaceuticals Ltd) |
| 66008 | Synjardy 12.5mg/1000mg tablets (Boehringer Ingelheim Ltd) |
| 7048 | Metformin 500mg modified-release tablets |
| 73285 | Metformin 500mg tablets (Waymade Healthcare Plc) |
| 73892 | Metformin 500mg tablets (Relonchem Ltd) |
| 11946 | Tolbutamide 50mg/ml Injection |
| 40365 | Glimepiride 1mg tablets (Actavis UK Ltd) |
| 47894 | Nazdol MR 30mg tablets (Consilient Health Ltd) |
| 60328 | Alogliptin 12.5mg tablets |
| 37617 | Rosiglitazone 2mg tablet |
| 33562 | Duclazide 80mg Tablet (Dumex Ltd) |
| 73673 | Metformin 500mg/5ml oral solution sugar free (Sigma Pharmaceuticals Plc) |
| 45831 | Dacadis MR 30mg tablets (Mylan) |
| 39988 | Metformin 500mg oral powder sachets sugar free |
| 63929 | Canagliflozin 50mg / Metformin 1g tablets |
| 59809 | Alogliptin 6.25mg tablets |
| 44304 | Glyconon 500mg Tablet (DDSA Pharmaceuticals Ltd) |
| 74963 | Romozin 200mg Tablet (Glaxo Wellcome UK Ltd) |
| 19472 | Actos 45mg tablets (Takeda UK Ltd) |
| 68636 | Metformin 850mg/5ml oral solution sugar free |
| 72052 | Metformin 500mg Tablet (Lagap) |
| 21832 | Diabetamide 5mg tablets (Ashbourne Pharmaceuticals Ltd) |
| 75987 | Semaglutide 0.5mg/0.37ml solution for injection 1.5ml pre-filled disposable device |
| 322 | HUMALOG injection 100 iu/ml [LILLY] |
| 547 | glipizide tablets 2.5mg |
| 1587 | MONOTARD injection 100 units/ml [NOVO] |
| 1594 | ACTRAPID NOVOLET 100 iu/ml [NOVO] |
| 1844 | ULTRATARD injection 100 units/ml [NOVO] |
| 2455 | MIXTARD 20 NOVOLET 100 iu/ml [NOVO] |
| 2456 | MIXTARD 10 NOVOLET 100 iu/ml [NOVO] |
| 2812 | MIXTARD 40 NOVOLET 100 iu/ml [NOVO] |
| 4093 | HUMULIN M2 injection 100 units/ml [LILLY] |
| 4129 | insulin soluble porcine injection 100 units/ml |
| 4247 | insulin isophane porcine injection 100 units/ml |
| 5059 | Novopen 3 CLASSIC insulin pen 3ml/2-70 units [NOVO] |
| 5164 | Novopen 3 demi insulin pen 3ml/1-35 units [NOVO] |
| 5214 | insulin lispro human prb injection 100 iu/ml |
| 5557 | Novopen 3 fun - blue insulin pen 3ml/2-70 units [NOVO] |
| 5649 | Autopen insulin pen 1.5ml/1-16 units [OWEN] |
| 6091 | Novopen 3 fun - red insulin pen 3ml/2-70 units [NOVO] |
| 6138 | Humapen Ergo burgundy insulin pen 3ml/1-60 units [LILLY] |
| 6470 | Autopen 24 insulin pen 3ml/2-42 units [OWEN] |
| 6554 | Autopen 24 insulin pen 3ml/1-21 units [OWEN] |
| 6753 | Novopen Junior green insulin pen 3ml/1-35 units [NOVO] |
| 6781 | Autopen Classic insulin pen 3ml/2-42 units [OWEN] |
| 6831 | Humapen Ergo teal insulin pen 3ml/1-60 units [LILLY] |
| 6855 | AVANDAMET tablets 2mg + 500mg [GLAXSK PHA] |
| 6981 | Novopen Junior yellow insulin pen 3ml/1-35 units [NOVO] |
| 7075 | Optipen Pro 1 green insulin pen 3ml/1-60 units [AVENTIS] |
| 7319 | MIXTARD 20 PENFILL injection suspension 100 units/ml [NOVO] |
| 7325 | AVANDAMET tablets 4mg + 1000mg [GLAXSK PHA] |
| 7537 | HUMULIN ZN injection 100 units/ml [LILLY] |
| 8390 | gliquidone tablets 30mg |
| 8841 | HUMULIN M5 injection 100 units/ml [LILLY] |
| 9565 | HUMAJECT S DISPOSABLE PEN injection solution 100 units/ml [LILLY] |
| 9618 | HYPURIN PORCINE 30/70 MIX injection 100 iu/ml [CP PHARM] |
| 9619 | BD Ultra Pen insulin pen 1.5ml [BECTON] |
| 9662 | AVANDIA tablets 4mg [GLAXSK PHA] |
| 10008 | mhi-500 needle free starter kit 10ml vial adaptor MH79002-01 [MED HOUSE] |
| 10009 | mhi-500 needle free starter kit 3ml vial adaptor MH79002-01A [MED HOUSE] |
| 10010 | mhi-500 3 month consumable kit size 6 nozzle, 10ml vial adaptor MH79002-07 [MED HOUSE] |
| 10011 | mhi-500 3 month consumable kit size 6 nozzle, 3ml vial adaptor MH79002-12 [MED HOUSE] |
| 10012 | mhi-500 3 month consumable kit size 7 nozzle, 10ml vial adaptor MH79002-08 [MED HOUSE] |
| 10013 | mhi-500 3 month consumable kit size 7 nozzle, 3ml vial adaptor MH79002-13 [MED HOUSE] |
| 10014 | mhi-500 10ml vial adaptor MH79002-09 [MED HOUSE] |
| 10015 | mhi-500 3ml vial adaptor MH79002-14 [MED HOUSE] |
| 10016 | mhi-500 nozzle size 6 MH79002-10 [MED HOUSE] |
| 10017 | mhi-500 nozzle size 7 MH79002-11 [MED HOUSE] |
| 10145 | Humapen Luxura insulin pen 3ml/1-60 units [LILLY] |
| 10175 | insulin isophane human pyr injection 100 iu/ml |
| 10244 | MIXTARD 40 PENFILL injection suspension 100 units/ml [NOVO] |
| 10245 | MIXTARD 10 PENFILL injection suspension 100 units/ml [NOVO] |
| 10547 | HUMULIN LENTE injection 100 units/ml [LILLY] |
| 11345 | BD Ultra Pen insulin pen 3.0ml [BECTON] |
| 11408 | Autopen Classic insulin pen 3ml/1-21 units [OWEN] |
| 12818 | MIXTARD 50 injection 50:50; 100 units/ml [NOVO] |
| 12897 | guar gum sachets 5g/sachet |
| 13096 | Autopen insulin pen 1.5ml/2-32 units [OWEN] |
| 13108 | Autopen Special Edition insulin pen 3ml/1-21 units [OWEN] |
| 13819 | HYPURIN PORCINE ISOPHANE injection 100 units/ml [CP PHARM] |
| 14164 | AVANDAMET tablets 2mg + 1000mg [GLAXSK PHA] |
| 14191 | Autopen Junior insulin pen 3ml/2-42 units [OWEN] |
| 14619 | insulin biphasic isophane porcine injection 30:70; 100 units/ml |
| 15232 | AVANDIA tablets 8mg [GLAXSK PHA] |
| 15294 | Innovo green insulin pen 3ml/1-70 units [NOVO] |
| 15484 | insulin isophane bovine injection 100 units/ml |
| 15895 | Innovo orange insulin pen 3ml/1-70 units [NOVO] |
| 15951 | Autopen Junior insulin pen 3ml/1-21 units [OWEN] |
| 17580 | AVANDAMET tablets 1mg + 500mg [GLAXSK PHA] |
| 17643 | Autopen Special Edition insulin pen 3ml/2-42 units [OWEN] |
| 17706 | MINODIAB tablets 2.5mg [PHARMACIA] |
| 18461 | insulin zinc suspension mixed human prb injection 100 units/ml |
| 18931 | insulin zinc suspension crystalline human prb - intermediate acting injection 100 units/ml |
| 19658 | GLURENORM tablets 30mg [SANOFI S] |
| 20634 | SQ-Pen 10ml vial adaptor pack SQ 005 [MED HOUSE] |
| 20635 | SQ-Pen nozzle pack SQ 002 [MED HOUSE] |
| 20636 | SQ-Pen starter pack SQ 001 [MED HOUSE] |
| 21422 | insulin biphasic isophane human cartridge injection suspension 40:60; 100 units/ml |
| 22697 | insulin biphasic isophane human pyr injection 50:50; 100 units/ml |
| 25735 | insulin biphasic isophane human cartridge injection suspension 20:80; 100 units/ml |
| 25736 | insulin biphasic isophane human cartridge injection suspension 10:90; 100 units/ml |
| 28851 | SQ-Pen 3 month 10ml consumable pack SQ 006 [MED HOUSE] |
| 31438 | SQ-Pen 3 month 3ml consumable pack SQ 004 [MED HOUSE] |
| 31439 | SQ-Pen 3ml vial adaptor pack SQ 003 [MED HOUSE] |
| 31465 | EXUBERA powder for inhalation 1mg [PFIZER] |
| 31467 | EXUBERA powder for inhalation 3mg [PFIZER] |
| 33232 | insulin biphasic isophane human crb injection 50:50; 100 units/ml |
| 35017 | Optipen Pro 1 white insulin pen 3ml/1-60 units [AVENTIS] |
| 35057 | OptiClik blue insulin pen 3ml/1-80 units [SANOFI/AVE] |
| 35078 | Optipen Pro 1 blue insulin pen 3ml/1-60 units [AVENTIS] |
| 35081 | OptiClik grey insulin pen 3ml/1-80 units [SANOFI/AVE] |
| 35218 | Optipen Pro 1 yellow insulin pen 3ml/1-60 units [AVENTIS] |
| 36355 | insulin human powder for inhalation 1mg |
| 36356 | insulin human powder for inhalation 3mg |
| 36430 | insulin soluble human disposable pen injection solution 100 units/ml |
| 37427 | Humapen Luxura HD insulin pen 3ml/1-30 units [LILLY] |

# Supplementary Table 3^21^ International cut off points for body mass index for overweight and obesity by sex between 2 and 18 years, defined to pass through body mass index of 25 and 30 kg/m^2^ at age 18, obtained by averaging data from Brazil, Great Britain, Hong Kong, Netherlands, Singapore, and United States

| **Age** | **Body mass index 25 kg/m^2^** | | **Body mass index 30 kg/m^2^** | |
| --- | --- | --- | --- | --- |
|  | **Males** | **Females** | **Males** | **Females** |
| 2 | 18.41 | 18.02 | 20.09 | 19.81 |
| 2.5 | 18.13 | 17.76 | 19.80 | 19.55 |
| 3 | 17.89 | 17.56 | 19.57 | 19.36 |
| 3.5 | 17.69 | 17.40 | 19.39 | 19.23 |
| 4 | 17.55 | 17.28 | 19.29 | 19.15 |
| 4.5 | 17.47 | 17.19 | 19.26 | 19.12 |
| 5 | 17.42 | 17.15 | 19.30 | 19.17 |
| 5.5 | 17.45 | 17.20 | 19.47 | 19.34 |
| 6 | 17.55 | 17.34 | 19.78 | 19.65 |
| 6.5 | 17.71 | 17.53 | 20.23 | 20.08 |
| 7 | 17.92 | 17.75 | 20.63 | 20.51 |
| 7.5 | 18.16 | 18.03 | 21.09 | 21.01 |
| 8 | 18.44 | 18.35 | 21.60 | 21.57 |
| 8.5 | 18.76 | 18.69 | 22.17 | 22.18 |
| 9 | 19.10 | 19.07 | 22.77 | 22.81 |
| 9.5 | 19.46 | 19.45 | 23.39 | 23.46 |
| 10 | 19.84 | 19.86 | 24.00 | 24.11 |
| 10.5 | 20.20 | 20.29 | 24.57 | 24.77 |
| 11 | 20.55 | 20.74 | 25.10 | 25.42 |
| 11.5 | 20.89 | 21.20 | 25.58 | 26.05 |
| 12 | 21.22 | 21.68 | 26.02 | 26.67 |
| 12.5 | 21.56 | 22.14 | 26.43 | 27.24 |
| 13 | 21.91 | 22.58 | 26.84 | 27.76 |
| 13.5 | 22.27 | 22.98 | 27.25 | 28.20 |
| 14 | 22.62 | 23.34 | 27.63 | 28.57 |
| 14.5 | 22.96 | 23.66 | 27.98 | 28.87 |
| 15 | 23.29 | 23.94 | 28.30 | 29.11 |
| 15.5 | 23.60 | 24.17 | 28.60 | 29.29 |
| 16 | 23.90 | 24.37 | 28.88 | 29.43 |
| 16.5 | 24.19 | 24.54 | 29.14 | 29.56 |
| 17 | 24.46 | 24.70 | 29.41 | 29.69 |
| 17.5 | 24.73 | 24.85 | 29.70 | 29.84 |
| 18 | 25 | 25 | 30 | 30 |

**Supplementary Table 4: Characteristics of diabetes cases in Down syndrome and population controls; percentages in brackets unless otherwise indicated.**

| **Variable** | **Category** | **Down syndrome** | **Population Controls** |
| --- | --- | --- | --- |
|  |  |  |  |
| **Total diabetes incidences** | | 287 | 1,254 |
|  |  |  |  |
| **Period** | 1990-9 | 15 (5) | 71 (6) |
|  | 2000-9 | 122 (43) | 550 (44) |
|  | 2010+ | 150 (52) | 633 (50) |
|  |  |  |  |
| **Sex** | Male | 137 (48) | 617 (49) |
|  | Female | 150 (52) | 637 (51) |
|  |  |  |  |
| **Age (years)** | Median (IQR) | 38 (28 to 49) | 53 (43 to 61) |
|  |  |  |  |
| **Type** | ‘Type 1’ | 37 (13) | 49 (4) |
|  | ‘Type 2’ | 250 (87) | 1,205 (96) |
|  |  |  |  |
| **BMI recorded^a^** |  | 218 (76) | 1,076 (86) |
|  |  |  |  |
| **BMI (Kg/m^2^)^a^** | Mean (SD) | 34.3 (8.7) | 33.2 (7.1) |
|  |  |  |  |
| **HbA1c recorded^b^** |  | 182 (63) | 960 (77) |
|  |  |  |  |
| **HbA1c (mmol/mol)^b^** | Median (IQR) | 53 (43 to 75) | 53 (45 to 75) |
|  |  |  |  |
| **Treatment in first 5 years from diagnosis** | Oral agents | 113 (39) | 722 (58) |
|  | Insulin | 58 (20) | 138 (11) |
|  |  |  |  |

^a^ maximum BMI record during five years before diagnosis

^b^ HbA1c values recorded up to 12 months after diagnosis

**Supplementary Table 5: Incidence of type 1 diabetes by sex, age-group and body mass index category for Down syndrome cases and population controls.**

|  |  | **Down syndrome** | | | **Population Controls** | | |
| --- | --- | --- | --- | --- | --- | --- | --- |
|  |  | **Type 1 diabetes diagnoses** | **Person years at risk** | **Incidence per 1,000 patient years (95% confidence interval)** | **Type 1 diabetes diagnoses** | **Person years at risk** | **Incidence per 1,000 patient years (95% confidence interval)** |
|  |  |  |  |  |  |  |  |
| **Total** |  | 37 | 83,160.5 | 0.44 (0.31 to 0.61) | 49 | 389,136.1 | 0.13 (0.09 to 0.17) |
|  |  |  |  |  |  |  |  |
| **Sex** | Male | 17 | 38,770.7 | 0.44 (0.26 to 0.70) | 26 | 190,023.8 | 0.14 (0.09 to 0.20) |
|  | Female | 20 | 44,389.8 | 0.45 (0.28 to 0.70) | 23 | 199,112.3 | 0.12 (0.07 to 0.17) |
|  |  |  |  |  |  |  |  |
| **Age-group** | 0-4 | 1 | 6,104.5 | 0.16 (0.004 to 0.91) | 3 | 33,986.7 | 0.09 (0.02 to 0.26) |
|  | 5-14 | 12 | 12,916.8 | 0.93 (0.48 to 1.62) | 21 | 65,148.9 | 0.32 (0.20 to 0.49) |
|  | 15-24 | 14 | 12,379.2 | 1.13 (0.62 to 1.90) | 10 | 56,126.1 | 0.18 (0.09 to 0.33) |
|  | 25-34 | 10 | 14,750.9 | 0.68 (0.33 to 1.25) | 15 | 52,598.1 | 0.29 (0.16 to 0.47) |
|  |  |  |  |  |  |  |  |
| **BMI category** | Underweight | 0 | 895.5 | - | 1 | 4,450.8 | 0.22 (0.01 to 1.25) |
|  | Normal weight | 7 | 12,408.2 | 0.56 (0.23 to 1.16) | 14 | 53,086.0 | 0.26 (0.14 to 0.44) |
|  | Overweight | 4 | 12,053.3 | 0.33 (0.09 to 0.85) | 7 | 41,988.1 | 0.17 (0.07 to 0.34) |
|  | Obese | 9 | 18,334.7 | 0.49 (0.22 to 0.93) | 9 | 39,601.2 | 0.23 (0.10 to 0.43) |
|  | Not recorded | 17 | 39,468.8 | 0.43 (0.25 to 0.69) | 18 | 250,010.0 | 0.07 (0.04 to 0.11) |
|  |  |  |  |  |  |  |  |

**Supplementary Table 6: Incidence of type 2 diabetes by sex, age-group and body mass index category for Down syndrome cases and population controls.**

|  |  | **Down Syndrome** | | | **Population Controls** | | |
| --- | --- | --- | --- | --- | --- | --- | --- |
|  |  | **Type 2 diabetes diagnoses** | **Person years at risk** | **Incidence per 1,000 patient years (95% confidence interval)** | **Type 2 diabetes diagnoses** | **Person years at risk** | **Incidence per 1,000 patient years (95% confidence interval)** |
| **Total** |  | 250 | 83,160.5 | 3.01 (2.65 to 3.40) | 1205 | 389,136.1 | 3.10 (2.92 to 3.28) |
|  |  |  |  |  |  |  |  |
| **Sex** | Male | 120 | 38,770.7 | 3.10 (2.57 to 3.70) | 591 | 190,023.8 | 3.11 (2.86 to 3.37) |
|  | Female | 130 | 44,389.8 | 2.93 (2.45 to 3.48) | 614 | 199,112.3 | 3.08 (2.84 to 3.34) |
|  |  |  |  |  |  |  |  |
| **Age-group** | 0-4 | 0 | 6,104.5 | - | 1 | 33,986.7 | 0.03 (0.00 to 0.16) |
|  | 5-14 | 8 | 12,916.8 | 0.62 (0.27 to 1.22) | 4 | 65,148.9 | 0.06 (0.02 to 0.16) |
|  | 15-24 | 20 | 12,379.2 | 1.62 (0.99 to 2.50) | 28 | 56,126.1 | 0.50 (0.33 to 0.72) |
|  | 25-34 | 47 | 14,750.9 | 3.19 (2.34 to 4.24) | 72 | 52,598.1 | 1.37 (1.07 to 1.72) |
|  | 35-44 | 77 | 16,366.0 | 4.70 (3.71 to 5.88) | 186 | 63,677.5 | 2.92 (2.52 to 3.37) |
|  | 45-54 | 61 | 13,704.9 | 4.45 (3.40 to 5.72) | 356 | 64,138.6 | 5.55 (4.99 to 6.16) |
|  | 55-64 | 32 | 6,006.0 | 5.33 (3.64 to 7.52) | 384 | 39,821.9 | 9.64 (8.70 to 10.66) |
|  | 65-75 | 5 | 932.1 | 5.36 (1.74 to 12.52) | 174 | 13,638.2 | 12.76 (10.93 to 14.80) |
|  |  |  |  |  |  |  |  |
| **BMI category** | Underweight | 4 | 895.5 | 4.46 (1.22 to 11.44) | 1 | 4,450.8 | 0.22 (0.01 to 1.25) |
|  | Normal weight | 24 | 12,408.2 | 1.93 (1.24 to 2.88) | 119 | 53,086.0 | 2.24 (1.86 to 2.68) |
|  | Overweight | 47 | 12,053.3 | 3.90 (2.87 to 5.19) | 299 | 41,988.1 | 7.12 (6.34 to 7.98) |
|  | Obese | 139 | 18,334.7 | 7.58 (6.37 to 8.95) | 669 | 39,601.2 | 16.89 (15.63 to 18.22) |
|  | Not recorded | 36 | 39,468.8 | 0.91 (0.64 to 1.26) | 117 | 250,010.0 | 0.47 (0.39 to 0.56) |

**Supplementary Figure 1: Incidence of type 1 diabetes (95% confidence interval) by age-group in DS (red) and controls (blue)**
**Supplementary Figure 2: Incidence of type 2 diabetes (95% confidence interval) by age-group in DS (red) and controls (blue)**
